# Effect Declines Are Systematic, Strong, and Ubiquitous: A Meta-Meta-Analysis of the Decline Effect in Intelligence Research

**DOI:** 10.3389/fpsyg.2019.02874

**Published:** 2019-12-19

**Authors:** Jakob Pietschnig, Magdalena Siegel, Junia Sophia Nur Eder, Georg Gittler

**Affiliations:** Department of Applied Psychology: Health, Development, Enhancement and Intervention, Faculty of Psychology, University of Vienna, Vienna, Austria

**Keywords:** decline effect, meta-meta-analysis, dissemination bias, effect misestimation, intelligence

## Abstract

Empirical sciences in general and psychological science in particular are plagued by replicability problems and biased published effect sizes. Although dissemination bias-related phenomena such as publication bias, time-lag bias, or visibility bias are well-known and have been intensively studied, another variant of effect distorting mechanisms, so-called decline effects, have not. Conceptually, decline effects are rooted in low initial (exploratory) study power due to strategic researcher behavior and can be expected to yield overproportional effect declines. Although decline effects have been documented in individual meta-analytic investigations, systematic evidence for decline effects in the psychological literature remains to date unavailable. Therefore, we present in this meta-meta-analysis a systematic investigation of the decline effect in intelligence research. In all, data from 22 meta-analyses comprising 36 meta-analytical and 1,391 primary effect sizes (*N* = 697,000+) that have been published in the journal *Intelligence* were included in our analyses. Two different analytic approaches showed consistent evidence for a higher prevalence of cross-temporal effect declines compared to effect increases, yielding a ratio of about 2:1. Moreover, effect declines were considerably stronger when referenced to the initial primary study within a meta-analysis, yielding about twice the magnitude of effect increases. Effect misestimations were more substantial when initial studies had smaller sample sizes and reported larger effects, thus indicating suboptimal initial study power as the main driver of effect misestimations in initial studies. *Post hoc* study power comparisons of initial versus subsequent studies were consistent with this interpretation, showing substantially lower initial study power of declining, than of increasing effects. Our findings add another facet to the ever accumulating evidence about non-trivial effect misestimations in the scientific literature. We therefore stress the necessity for more rigorous protocols when it comes to designing and conducting primary research as well as reporting findings in exploratory and replication studies. Increasing transparency in scientific processes such as data sharing, (exploratory) study preregistration, but also self- (or independent) replication preceding the publication of exploratory findings may be suitable approaches to strengthen the credibility of empirical research in general and psychological science in particular.

## Introduction

The credibility of results from empirical research in general and psychological science in particular depends on the reproducibility of published results. However, many, oftentimes spectacular, effects have turned out to be surprisingly hard to replicate (e.g., Ioannidis, 2005), raising concerns about a potential reproducibility crisis in psychology (Baker, 2016). Although credibility concerns may not necessarily be attributable to the increasing adoption of questionable research practices or publication pressure (Fanelli, 2018), the awareness and concerns about biased effects in the literature seems to have substantially increased since the proclamation of the most recent replication crisis.

Leaving some disconcerting but isolated cases of fraudulent studies and data analyses aside (e.g., the Stapel case; Levelt et al., 2012), the reproducibility crisis seems to be rooted in more insidious mechanisms than data fabrication and science fraud only. Although self-admittance rates of fraudulent practices are low (but not nil; see, Fiedler and Schwarz, 2016), other research practices and researcher behaviors that cause effect misestimation (i.e., systematic over- vs. underestimation) may play a more important role: Strategic research and submission behaviors, selective reporting, and *p*-hacking (see e.g., Bakker et al., 2012; Simonsohn et al., 2014) are only some of the mechanisms that may lead to a disproportionate number of biased effects in the scientific literature.

Importantly, such behaviors appear to be quite common, as self-admission rates and prevalence estimates suggest that the majority of researchers in psychology have engaged in at least one of these questionable research practices (e.g., John et al., 2012). A recent review of 64 studies that addressed this topic showed that evidence for such practices almost invariably emerged, regardless of the investigated subdiscipline (Banks et al., 2016).

Systematic large-scale assessments indicate that only about 40% of psychological experiments replicate (Open Science Collaboration, 2015). Importantly, replicability problems and biased estimates do not seem to be an exclusive problem of psychological science, but plague other empirical disciplines as well (e.g., biology: Begley and Ellis, 2012; genetics: Siontis et al., 2010). Such observations have led some critical voices to assert that most effects that are reported in the empirical literature are inflated (Ioannidis, 2008). This is important because inflated effects can pollute a field for a considerable amount of time, leading replicators of inflated (or outright false) effects to base their efforts on faulty assumptions.

Indeed, several forms of dissemination bias, such as publication, time-lag, or visibility bias, have been linked to effect inflation in the past (e.g., Rothstein et al., 2005). Another proposed mechanism that may help in understanding effect misestimations is the so-called *decline effect*, which describes decreases in the strength of observed effect sizes as evidence accumulates over time. Cross-temporal observations of the decline effect have been frequently made on a meta-analytic level but a systematic (i.e., meta-meta-analytic) investigation of this phenomenon is still lacking.

Examples of declining effects that have been observed in the psychological literature include declining correlation strengths between IQ and *in vivo* brain volume (Pietschnig et al., 2015) or shrinking beneficial effects of listening to music on spatial task performance (i.e., the Mozart effect; Pietschnig et al., 2010). Conceptually, the former case represents an *inflated decline effect* (i.e., effect sizes that are reported are inflated, although a true, albeit smaller, effect exists) whilst the latter case illustrates most likely a certain type of a *false positive decline effect* (i.e., the initially found positive effect represents an artifact; see Protzko and Schooler, 2017, for an overview about the decline effect). Both of these examples have in common, that the first reported effect in the literature investigating this research question (i.e., the initial or exploratory finding) was substantially larger than virtually all subsequent replication effects. However, evidence for the decline effect has also been documented in other empirical disciplines such as the medical sciences (e.g., Lau et al., 1995).

Obviously, empirical research is inherently susceptible to chance findings. Therefore, individual studies may be expected to yield inflated effect estimates in some cases and deflated in others (i.e., representing the well-known mechanism of regression to the mean). On the one hand, unsystematic effect inaccuracies due to mere error variance should lead to an about equal number of effect increases and declines over time and do not pose a threat to empirical research as a whole. Systematic effect misestimations in terms of overproportional declines (or increases), on the other hand, do. Should, however, certain research practices and mechanisms favor one type of time-trend over the other (i.e., cross-temporal declines vs. increases), this type of time-trend will be overrepresented in the available literature, regardless of the investigated research question. We argue that due to publication-related mechanisms and strategic researcher behaviors, effect declines should be more prevalent in the literature than effect increases.

On the one hand, whilst dissemination biases may be expected to be driven by questionable research practices for a large part (i.e., in cases where the publication of results is not suppressed altogether, but data are *p*-hacked, selectively reported, peeked at, or similar; for an overview see Wicherts et al., 2016), the decline effect is mainly rooted in low exploratory study power. On the other hand, the trajectory of the decline subsequent to the publication of an inflated initial result may be very well expected to be related to various types of bias. Because inflated initial effects anchor the expectations of replicators to a certain effect strength, the power of replication studies may often be insufficient to detect a reasonable estimate of the true effect (i.e., only observations of inflated effects become significant). Such unsuccessful replications are in turn more likely to be either left unpublished or *p*-hacked until a desirable result is obtained. In other words, this may mean that whilst decline effects are triggered by low power, dissemination biases and questionable research practices may shape the subsequent decline trajectory.

Specifically, it has been well-established that limited resources and funding in combination with publication pressure incentivize submission and publication of significant, strong, and surprising effects (e.g., Ioannidis, 2008). This makes primary research, particularly exploratory research, inherently risky, because (exploratory) hypotheses may be wrong and therefore yield non-significant results. Non-significant results, however, may be costly for researchers, because publication expectancies, especially in renowned journals (regardless of whether these expectancies occur on journal, reviewer, or author level; see Ferguson and Heene, 2012), are lower for null-findings and may be therefore disadvantageous to a researcher’s career.

Therefore, researchers investigating an exploratory hypothesis may opt to minimize their risk of obtaining non-significant and consequently potentially career-inhibiting results by distributing their resources into investigating multiple hypotheses, thus maximizing the probability of obtaining a desirable (and highly publishable) result in at least one of the investigations. Because researcher resources are typically limited, multiple investigations (and therefore sampling efforts) will likely lead to smaller within-investigation sample sizes and consequently lower study power. In fact, systematic evidence from renowned journals in psychological science indicate median total sample sizes ranging from 40 to 48 for bivariate tests (Wetzels et al., 2011; Anderson et al., 2017). These strategic research behaviors therefore do not only maximize the publication probability, but also the within-study error variance.

In such scenarios (i.e., small sample sizes and large error variances), observed effects need to be comparatively large in order to become significant. This mechanism, combined with strategic submission behavior of researchers, increases the likelihood of the publication of inflated effects compared to more accurate or deflated effects. In other words, sample sizes of primary studies may be often inadequate to detect the true effect by means of conventional null hypothesis significance testing which leads to more frequent publication of inflated effects and subsequent declines.

Because effect misrepresentations should be largely unrelated to topical specifics (i.e., the research question) but may conceptually be deemed to be a function of low study power, we aimed at investigating a psychological subdiscipline that possesses comparatively large average study power. The reason for this approach was that well- (or at least not strikingly suboptimally-) powered subdisciplines can be expected to lead to conservative estimates of effect misestimation prevalence and strength. If effect declines outnumber increases in a comparatively well-powered field, this is suggestive of even higher prevalences of effect declines in other fields that are characterized by lower power averages. For this reason, intelligence research appeared to be particularly suitable because this field is characterized by large-scale studies and yields larger average power estimates than psychology on the whole (Pietschnig et al., 2018).

In the present meta-meta-analysis, we therefore aimed at investigating cross-temporal changes of effect sizes in all meta-analyses that have been published in the journal *Intelligence*, which is the flagship journal in this field and can be expected to include a meaningful cross-section of intelligence research. This journal has been established in 1977, which almost coincides with the publication of the earliest modern meta-analytical application (i.e., Glass, 1976) and therefore was expected to provide a comparatively broad range of potentially includable meta-analyses. Based on two indicators of effect change (crude differences between the first published effect size and the summary effect as well as regression slopes; see section “Statistical Analyses”), we expected to observe (i) more and (ii) stronger declining than increasing effects, as well as (iii) stronger evidence for publication bias in meta-analyses that are associated with declining than those that are associated with increasing effects. Furthermore, we examine influences of initial (exploratory) study characteristics (sample size, effect strength, citation numbers, journal impact factor, and publication year) and meta-analytic summary effect strength on effect misestimations.

Strategic submission behaviors favor high-impact publications of strong effects and unexpected results which are more likely to be cited and may be more frequently observed in smaller samples. However, results from data of small samples are less accurate and may lead to considerable effect misestimations due to large error variance. Therefore, we expected initial effect strengths, citation numbers, and impact factors to be positively, but sample sizes to be negatively related to effect misestimation. Because large true effects are arguably even more prone to be detected with an underpowered design (but will in such a case yield inflated results), we expected positive associations between meta-analytical summary effects and misestimations. Moreover, because publication pressure is likely to have increased in the past decades, we explored potential positive relations with initial study publication years.

## Materials and Methods

### Literature Search

We searched the online database ISI Web of Knowledge for the term “meta-analy^∗^” (topic) in the journal *Intelligence* (publication name) up to July 18, 2018. We deemed this strategy appropriate because the journal *Intelligence* has been completely indexed in this online database, which consequently enabled us to retrieve all meta-analyses that have been published in this journal.

### Inclusion Criteria

Meta-analyses were suitable for inclusion if they fulfilled the following three criteria. First, meta-analyses had to report traditional effect sizes (i.e., Cohen *d*, Hedges *g*, Pearson *r*, Odds ratios, Log Odds ratios, or Fisher *z*) rather than effect sizes that had been obtained through secondary analyses, such as the Method of Correlated Vectors (e.g., Armstrong et al., 2014). This was deemed appropriate because results of such secondary analyses should not be subject to effect biasing mechanisms. Similarly, effects from cross-temporal meta-analyses (e.g., Pietschnig and Gittler, 2015) were not included, because the publication of unobtrusively collected mean values in primary studies are not expected to be confounded by dissemination bias. In other words, cross-temporal meta-analyses do not rely on synthesizing effect sizes but on aggregating mean values which are not expected to be affected by decline effects.

Second, included meta-analyses should not have been arbitrarily limited in terms of the time frame of the literature search. Specifically, if the literature search had been confined to a specific number of years, a justification had to be provided which explained the time limitation based on content-specific reasons (e.g., introduction of a novel conceptualization or assessment method). Third, the effect size of the initial study had to be either reported in the meta-analysis or had to be identifiable based on the primary paper that it had been reported in. Finally, to be eligible for our cross-temporal calculations of regression slopes, data of all primary studies that had been included within a given meta-analysis had to be provided either in data tables or online Supplementary Materials.

### Coding

Full texts of all search hits were obtained and coded twice into categories by the same experienced researcher (JP). Summary effects as reported by the authors of a given meta-analysis, effect size metric, number of included samples, total sample sizes, initial study sample sizes, initial study effect size, initial study citation numbers, and the 2017 Impact Factors of the journals that an initial study had been published in were recorded. Discrepancies between the first and second coding run were resolved through discussion with another independent researcher (MS). A flowchart of study inclusion and study references including reasons for exclusion can be found in the Supplementary Figure S1 and Supplementary Data Sheet S2.

### Statistical Analyses

Prior to all calculations, effect sizes were transformed into Fisher *z* to obtain a common effect metric. We used two analytic approaches to quantify effect changes (i.e., declines vs. increases). On the one hand, we calculated crude differences between the absolute initial study effects and their corresponding meta-analytical summary effects, in cases where signs of effect sizes yielded an identical direction. In cases where initial and summary effects yielded different signs (i.e., indicating a so-called proteus phenomenon, which can be understood as an extreme case of the decline effect; e.g., Pfeiffer et al., 2011), we summed up absolute initial and summary effect sizes. This procedure resulted in either positive crude differences (indicating declining effects) or negative crude differences (indicating increasing effects). For ease of interpretation, effect sizes were transformed into the Pearson *r* metric prior to reporting.

On the other hand, we calculated single precision-weighted (i.e., according to the inverse variances of primary studies) mixed-effects meta-regressions of effect sizes of primary studies on publication year to obtain the regression slope (i.e., representing effect declines or increases). Mixed-effects meta-regressions correspond broadly to conventional regression analyses but treat effect sizes as dependent variables and contain elements of both fixed-effect (i.e., in terms of the predictor) and random-effects models (i.e., in terms of the residual heterogeneity; Viechtbauer et al., 2015). Of note, we could only perform these calculations when data of primary studies that were included in a respective meta-analysis had been reported. The resulting regression slopes were interpreted as evidence for effect declines if their signs did not match the sign of the initial study effect and as evidence for increases if their signs matched the initial effect sign. Proteus effects (i.e., cases where initial and summary effect signs did not match) were treated as a separate subgroup in our analyses. In subsequent analyses, crude differences and regression slopes were used as dependent variables.

In our meta-meta-analysis, we first calculated average crude differences and regression slopes in terms of overall effect deviations, as well as subgrouped according to declines, increases, and proteus effects by means of random-effects and fixed-effect models (weighted by number of samples minus three within each meta-analysis). In the random-effects model, it is assumed that observed effect sizes from individual studies (presently: crude differences or slopes) represent estimates of effects that originate from different effect size distributions, thus necessitating consideration of systematic between-studies variance for effect size calculations (i.e., weights of individual observations are less influential and confidence intervals increase). In fixed-effect models, all observed effect sizes are considered to represent estimates of a single true effect (i.e., they are elements of a single distribution) which means that between-studies heterogeneity is attributed to sampling error (i.e., weights of individual observations are more influential and confidence intervals narrow; for a detailed explanation, see Borenstein et al., 2009).

By means of a graphical approach which broadly resembles (recursive) cumulative meta-analytical approaches as summarized elsewhere (Banks et al., 2012; Koricheva et al., 2013), we meta-meta-analytically illustrated continuous effect changes over time. In this approach, we cumulatively calculated summary effect sizes according to primary study publication years (if more than one study had been published in any given year, the individual effect sizes were synthesized by means of random-effects models prior to cumulation) and referenced these effect changes to the initial study effect that had been constrained to zero. The resulting lightning plot visualizes effect developments in terms of number (i.e., increases vs. declines) and strength of changes in the *z*-metric.

Second, we examined potential influences of initial study characteristics as well as the meta-analytical summary effect on the strength of effect changes over time (i.e., crude differences and regression slopes). In a series of single precision-weighted mixed-effects and fixed-effect meta-regressions as well as unweighted fixed-effect meta-regressions, we investigated influences of (i) initial study sample sizes, effect sizes, absolute and annual citation numbers, 2017 impact factors of the journals that an initial study had been published in, as well as (ii) the strength of meta-analytical summary effects on both effect misestimations in general and effect declines in particular.

Third, following a procedure broadly similar to that of Button et al. (2013), we calculated the average power of primary studies within each meta-analysis to detect the respective summary effect. To do so, we first recalculated meta-analytical summary effects based on the available data by means of random-effects models. Subsequently, we used the resulting summary effect estimate as well as the reported sample sizes of primary studies to calculate the power of each primary study (a common alpha level of 0.05 was assumed for two-tailed tests). This procedure allowed us to assess how much power the primary studies within each meta-analysis on average had to detect the respective observed summary effect size.

Finally, when data of primary effect sizes were available, we applied eight different methods to assess indications of potential dissemination bias within the individual meta-analyses. Importantly, different types of dissemination bias detection methods are not equally sensitive or suitable to provide information about different types of bias. For instance, funnel plot asymmetry-based assessments such as trim-and-fill are not able to detect *p*-hacking whilst approaches that can (such as *p*-uniform or *p*-curve) are more vulnerable to distributional characteristics of effect sizes than regression approaches (for an overview about different bias types and detection methods, see Simonsohn et al., 2014). So whilst no method has remained uncriticized so far and the application of any method in isolation may prove suboptimal in regard to one aspect or another, using a mix of different modern assessment methods has been shown to be the most useful approach to reduce bias (Carter et al., 2019).

Consequently, we used the trim-and-fill method (Duval and Tweedie, 2000), Begg and Mazumdar’s rank correlation test (Begg and Mazumdar, 1994), Sterne and Egger’s regression approach (Sterne and Egger, 2005), excess significance testing (Ioannidis and Trikalinos, 2007), *p*-uniform (Simonsohn et al., 2014), *p*-curve (van Assen et al., 2015), a selection model approach (Vevea and Woods, 2005), and PET-PEESE (Stanley and Doucouliagos, 2014). In accordance with common guidelines, individual methods were considered to be indicative of publication bias if either (i) *p* was smaller than 0.05 (*p*-uniform) or 0.10 (Begg and Mazumdar’s rank correlation test, Sterne and Egger’s regression, excess significance testing), (ii) the half-curve *p*-value was larger than 0.05 or both half- and full-curve *p*-values were larger than 0.10 (*p*-curve), or (iii) the difference between the adjusted estimate and the meta-analytic estimate was larger than 20% of the meta-analytic estimate in any (trim-and-fill) or a negative direction (moderate one-tailed selection of Vevea and Woods’ selection models and PET-PEESE; see, Banks et al., 2018). Following well-established procedures, we interpreted for PET-PEESE the PET estimates when *p*-values exceeded 0.10 and PEESE estimates in all remaining cases (Stanley, 2017).

All analyses were performed by means of the open source software R (R Core Team, 2017), specifically the packages *metafor* (Viechtbauer, 2010), *puniform* (van Aert, 2018), and *pwr* (Champely, 2018). We interpret our results according to the well-established classification of effect sizes by Cohen (1988).

### Final Sample

In all, 22 meta-analyses met our inclusion criteria yielding *k* = 36 meta-analytical effect sizes (1,391 primary study effect sizes; *N* = 697,639) with 24 reporting Pearson *r*, 10 Cohen *d*, and 2 Hedges *g* effect metrics. Moreover, primary study data were available from 18 studies (*k* = 29; 991 primary study effect sizes; *N* = 373,186), which allowed us to recalculate summary effects, analyze the average primary study power, and assess dissemination bias.

## Results

**FIGURE 1:**
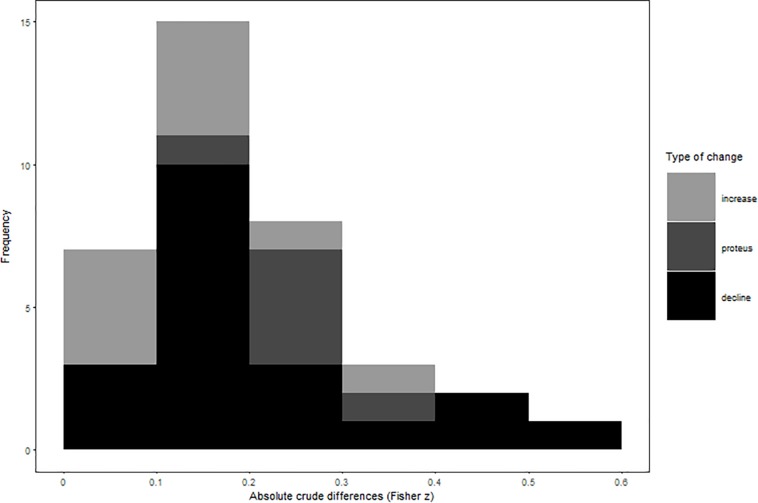
Absolute values of effect misestimations by type of change.

In all our analyses, we focus our interpretation of results on weighted random- and mixed-effects models, but provide additional results of unweighted and fixed-effect calculations in our Tables 1 and 2. Our results showed, that initial study effects misestimated observed summary effects by a small-to-moderate effect size regardless of their direction (crude difference in Pearson *r* = 0.17; see Figure 1 and Table 1 for effect misestimations by type). Crude effect declines outnumbered effect increases at a ratio of 2:1 and were considerably stronger than increases (absolute crude differences in Pearson *r* = 0.18 vs. 0.08, respectively). Moreover, six meta-analyses showed a proteus effect, indicating inconsistent effect signs of initial study and meta-analytical summary effects. As expected, crude differences were strongest for proteus effects, yielding average initial study effect size misestimations of Pearson *r* = 0.26.

**TABLE 1:** Average deviations from the summary effect according to crude differences and regression coefficients.

	**Crude differences**				**Regression slopes**			
	***k***	***N***	***r* (RE)**	***r* (FE)**	***k***	***N***	***r* (RE)**	***r* (FE)**
Overall	36	697639	0.167^∗∗∗^	0.168^∗∗∗^	29	373186	0.009	0.009
Declines	20	400869	0.180^∗∗∗^	0.207^∗∗∗^	15	164557	0.010	0.010
Increases	10	219390	0.076	0.075	9	159222	0.007	0.007
Proteus	6	77380	0.262^∗∗^	0.262^∗∗^	5	49407	0.017	0.017

**k*, number of meta-analyses; *N*, number of individual participants; RE, random-effects model; FE, fixed-effect model. ^∗∗^*p* < 0.01; ^∗∗∗^*p* < 0.001.*

**TABLE 2:** Single meta-regressions of initial study characteristics and meta-analytic summary effects on crude differences and regression coefficients.

	**Overall**							**Declines**						
			**Weighted**	**Weighted**		**Unweighted**				**Weighted**	**Weighted**		**Unweighted**	
	***k***	***N***	**slope (ME)**	**slope (FE)**	**η^2^**	**slope (FE)**	**η^2^**	***k***	***N***	**slope (ME)**	**slope (FE)**	**η^2^**	**slope (FE)**	**η^2^**
**Crude differences**														
Initial *n*	34	554053	> −0.001	> −0.001	0.04	> −0.001	0.05	18	293869	> −0.001	> −0.001	0.05	> −0.001	0.01
Initial effect size	36	697639	0.316^∗^	0.303	0.19	0.257	0.23	20	366618	0.779^∗∗∗^	0.721^∗∗∗^	0.69	0.526^∗∗∗^	0.61
Initial citation numbers	34	613308	<0.001	<0.001	<0.01	<0.001	<0.01	19	330278	> −0.001	> −0.001	0.08	> −0.001	0.04
Initial annual citation numbers	34	613308	<0.001	0.001	<0.01	> −0.001	<0.01	19	330278	–0.001	–0.002	0.04	–0.001	0.03
Initial 2017 impact factor	20	356396	0.005	0.005	0.13	0.004	0.15	8	114851	0.003	0.003	0.12	0.004	0.17
Initial study publication year	36	697639	–0.008	–0.001	0.01	–0.001	0.02	20	366618	–0.003	−0.003^∗^	0.30	–0.002	0.12
Summary effect	36	697639	–0.087	–0.297	0.07	0.121	0.02	20	366618	–0.186	–0.379	0.06	0.003	<0.01
**Regression coefficients**														
Initial *n*	29	373186	> −0.001	> −0.001	0.02	> −0.001	0.04	15	164557	> −0.001	> −0.001	0.11	> −0.001	0.09
Initial effect size	29	373186	–0.022	–0.022	0.08	–0.005	<0.01	15	164557	0.010	0.010	0.01	0.010	0.01
Initial citation numbers	27	288855	<0.001	<0.001	0.03	> −0.001	<0.01	14	93966	> −0.001	> −0.001	0.01	> −0.001	0.01
Initial annual citation numbers	27	288855	<0.001	<0.001	0.02	> −0.001	<0.01	14	93966	> −0.001	> −0.001	0.01	> −0.001	0.02
Initial 2017 impact factor	17	195965	<0.001	<0.001	<0.01	> −0.001	<0.01	7	42561	> −0.001	> −0.001	<0.01	<0.001	<0.01
Initial study publication year	29	373186	<0.001	<0.001	0.28	< 0.001^∗^	0.15	15	164557	<0.001	<0.001	0.11	<0.001	0.10
Summary effect	29	373186	–0.008	–0.008	0.01	0.018	0.03	15	164557	0.056	0.056	0.10	0.06	0.14

**k*, number of meta-analyses; *N*, number of individual participants; FE, fixed-effect model; ME, mixed-effects model; all η^2^ values are based on fixed-effect models. ^∗^*p* < 0.05, ^∗∗∗^*p* < 0.001.*

**FIGURE 2:**
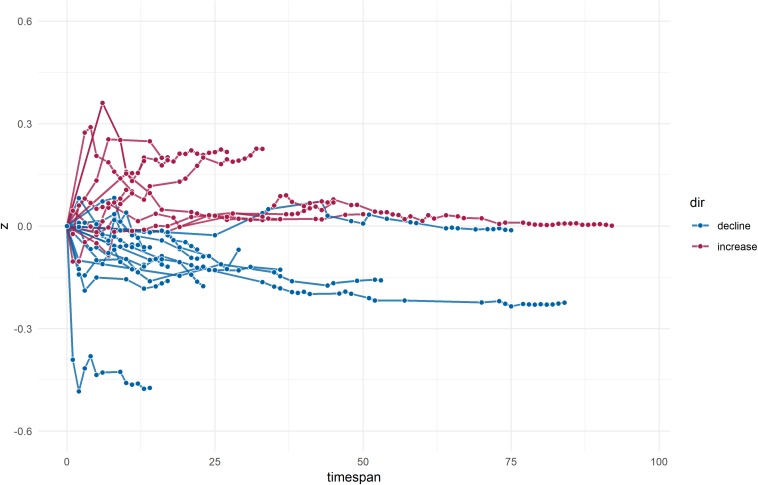
Lightning plot of cumulative effect changes over time. Proteus effects are not shown; dots within trajectories indicate the effect change in reference to the previous observed estimate (i.e., initial study effect or previous dot, respectively).

Analyses of absolute regression slopes indicated annual effect changes of about *r* = 0.01 (i.e., indicating expectable effect changes of *r* = 0.10 per decade as referenced to the initial study effect) regardless of the trajectory sign. Consistent with the results of crude differences, analyses of regression coefficients showed more prevalent (roughly maintaining a 2:1 ratio) and stronger effect declines than increases. Once more, cross-temporal effect changes were strongest for meta-analyses that showed a proteus effect (numerical results are detailed in Table 1). Trajectories of cumulative declines and increases over time (relative to the earliest published effect size) are illustrated in Figure 2.

For crude declines, examination of potential moderating variables yielded non-trivial and hypothesis-conforming positive associations of effect misestimations with initial effect size strength and initial study 2017 impact factors as well as negative associations with initial study sample sizes (η^2^s = 0.69, 0.12, and 0.05, respectively). The summary effect strength and – in contrast to our expectations – annual initial study citation numbers as well as publication year were negatively associated with effect misestimations (η^2^s = 0.06, 0.04, and 0.30, respectively, see top right section of Table 2).

Moderator analyses for overall effect changes (i.e., regardless of their direction) were largely consistent with results from crude declines but showed on the whole weaker effects. Once more, initial effect size strength and initial study 2017 impact factors were positively and initial study sample sizes were negatively associated with effect estimations (η^2^s = 0.19, 0.13, and 0.04, respectively). Associations of misestimations with summary effects were negative (η^2^ = 0.07) and annual initial study citation numbers showed the expected (albeit trivial) positive sign. Once more, associations with initial study publication year were negative (η^2^ = 0.01; see top left section of Table 2).

Effects of moderators on regression slopes for both overall misestimations and declining effects were smaller than effects on crude differences and less consistent between subgroups. Sample size of the initial study showed a consistent non-trivial negative association with overall effect misestimations and declines (η^2^s = 0.02 and 0.11). Interestingly, initial study publication year showed the expected non-trivial positive association with effect misestimations for both overall and declining effects (η^2^s = 0.28 and 0.11). All other predictors showed inconsistent signs between overall and decline analyses, yielding no clearly interpretable pattern (for details see bottom of Table 2). We repeated all above analyses for annual citation numbers with winsorized values (lower and upper thresholds were assumed according to the 10th and 90th percentile) because of large outliers, but obtained virtually identical results (numerical values omitted).

**FIGURE 3:**
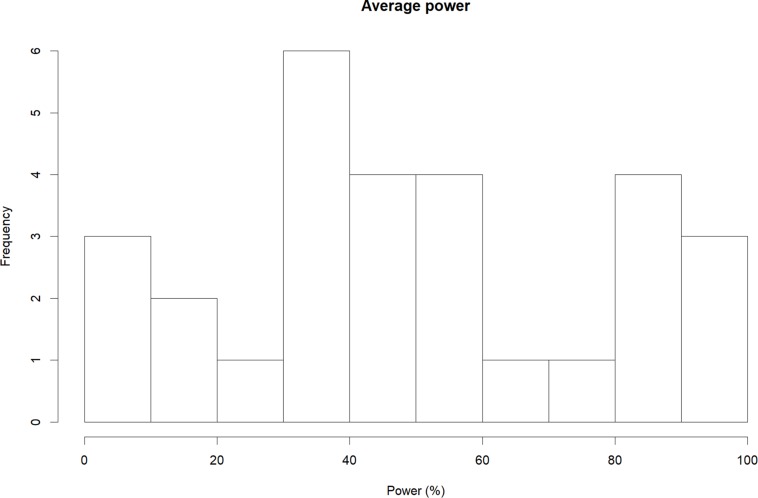
Average power of primary studies in 29 meta-analytic data sets that primary study data were available for.

Results of our power analyses showed that the average primary study had 50.24% power (*Md* = 48.18%; *k* = 29; Figure 3) to detect the respective summary effect. The most adequately powered studies were observed for meta-analyses that showed cross-temporal increases (mean = 59.76%; *Md* = 51.79%; *k* = 9), followed by declines (mean = 52.31%; *Md* = 48.18%; *k* = 15), and proteus effects (mean = 26.90%; *Md* = 10.58%; *k* = 5). Interestingly, although the average power of initial studies was similar in size compared to all other primary studies (mean = 48.46%; *Md* = 45.05%; *k* = 29), subgrouping according to effect changes yielded noticeable systematic differences: Specifically, the power of initial studies that belonged to increasing effects (mean = 66.05%; *Md* = 87.46%; *k* = 9) was markedly larger than the power of studies belonging to declining (mean = 48.85%; *Md* = 45.05%; *k* = 15) or proteus effects (mean = 15.65%; *Md* = 12.00%; *k* = 5).

In terms of dissemination bias indication, bias sensitivity varied considerably between methods (descriptive statistics of bias indication methods are provided in Table 3). However, visual inspection of within-meta-analysis bias indication frequencies did not suggest meaningful differences between meta-analyses that showed effect declines and those that showed increases (Table 4).

**TABLE 3:** Frequencies of bias indication within meta-analyses by method.

		**Begg and Mazumdar’s**	**Sterne and**	**Excess**				
	**Trim-and-fill**	**rank correlation**	**Egger regression**	**significance test**	***p*-uniform**	***p*-curve**	**Selection models**	**PET-PEESE**
**Crude differences (*k* = 29)**								
Declines	1(7%)	2(13%)	4(27%)	1(7%)	1(7%)	0(0%)	4(27%)	2(13%)
Increases	1(11%)	1(11%)	2(22%)	0(0%)	2(25%)	0(0%)	3(33%)	3(33%)
Proteus	3(60%)	0(0%)	2(40%)	0(0%)	0(0%)	0(0%)	2(40%)	2(40%)
**Regression coefficients (*k* = 24)**								
Declines	1(7%)	1(7%)	2(13%)	1(7%)	2(13%)	0(0%)	5(33%)	2(13%)
Increases	1(11%)	2(22%)	4(44%)	0(0%)	1(12%)	0(0%)	2(22%)	3(33%)

*Cell entries indicate absolute (and relative) frequencies of bias indication; *k* = 29/24, excepting *p*-uniform (*k* = 27/23), and *p*-curve (*k* = 26/23). *k* Declines = 15, *k* Increases = 9, *k* Proteus = 5.*

**TABLE 4:** Number of methods that were indicative of bias within meta-analyses according to effect direction.

	**0**	**1**	**2**	**3**	**4**
**Crude differences (k = 29)**					
Declines	6(40%)	5(33%)	2(13%)	2(13%)	0(0%)
Increases	2(22%)	4(44%)	2(22%)	0(0%)	1(11%)
Proteus	1(20%)	2(40%)	0(0%)	1(20%)	1(20%)
**Regression coefficients (k = 24)**					
Declines	6(40%)	6(40%)	2(13%)	0(0%)	1(7%)
Increases	2(22%)	3(33%)	2(22%)	2(22%)	0(0%)

**k* Declines = 15, *k* Increases = 9, and *k* Proteus = 5. Within-row percentages refer to absolute study number of the respective effect trajectory.*

Sensitivity analyses showed that bias indications (i.e., meta-analyses in which at least one method showed bias indications) did not appear to be driven by outlier-related between-studies heterogeneity: Neither *I*^2^ values nor maximum differences between *I*^2^ estimates of leave-one-out analyses in the individual meta-analyses showed significant differences between meta-analyses with bias and those without bias indication (independent *t*-tests yielded *p*s = 0.460 and 0.736; Cohen *d*s = 0.28 and 0.14, respectively). Moreover, maximum *I*^2^ differences in leave-one-out analyses within individual meta-analyses were similar in strength for meta-analyses with and those without bias indications, showing maximum *I*^2^ changes of about 36% and 30%, respectively. Influence diagnostics (Viechtbauer and Cheung, 2010) for individual meta-analyses showed that these sensitivity analyses were suitable for robustness assessments of our analyses because most meta-analyses did not show any (*k* = 18; 62.07%) or only one outlier (*k* = 7; 24.13%; *k* = 4 analyses showed 2 + outliers). Detailed results of influence diagnostics are provided in the Supplementary Data Sheet S1.

## Discussion

In this meta-meta-analysis, we show that effect declines over time systematically outnumber effect increases at a ratio of about two to one. Moreover, besides from being more prevalent, effect declines seem to be substantially stronger than effect increases. This observation has important implications for both designing and reporting primary (particularly exploratory) studies, as well as for interpreting novel (exploratory) effects from such studies. Although we examined these effects based on (diverse) research questions within the framework of intelligence research, it seems reasonable to assume that the presently discussed mechanisms may not only apply to research questions in this field, but rather to psychological science on the whole and empirical science in general.

First, our results indicate that regardless of the investigated exploratory hypothesis and the direction of a subsequent temporal change, initial findings misestimate the true effect on average by a small-to-moderate effect. Although the observation that exploratory effects are not always numerically entirely accurate representations of the true effect certainly does not come as a surprise, the extent of this inaccuracy might do so. In fact, median effect sizes that are usually reported in the individual differences research literature have been shown not to be substantially stronger than the presently typically observed effect misestimation: While typical effect strengths average about *r* = 0.19 (see Gignac and Szodorai, 2016), we presently observed average misestimations of about *r* = 0.17.

Second, crude effect declines over time are more prevalent and stronger than effect increases. This seems even more important when considering that we observed positive associations of effect declines with initial effect sizes, indicating stronger declines of stronger initial effects. These observations are consistent with the assumption that the decline effect is ultimately rooted in biased reporting due to suboptimally powered effects that originate from questionable research practices and strategic researcher behaviors. This interpretation is further supported by the observed relationship between the meta-analytical summary effects and the average sample sizes in the respective meta-analysis. The average reported effect of *r* = 0.19 in individual differences research (Gignac and Szodorai, 2016) requires a sample size of more than 200 participants in order to become significant when assuming 80% power for a two-tailed bivariate test. In the present study we observed a mean initial study sample size of *n* = 127 (*Md* = 116) for declining effects, but a mean summary effect around *r* = 0.12. This means that the average observed initial sample was too small to detect the average summary effect.

In the light of the observed low average study power (here: 50.24%), it seems unsurprising that effect misestimations of primary and particularly initial studies are substantial. The differentiated patterns of true effect misestimations – yielding largest crude differences for the proteus effects (which can be interpreted as extreme cases of decline effects), substantial crude differences for decline effects, and less severe ones for effect increases – are consistent with low (initial) study power and strategic researcher behavior as causes of the decline effect. In this vein, it needs to be noted that the presently observed suboptimal average study power does not seem to be unique to intelligence research. In fact, the available evidence from other subdisciplines in psychology yields even less favorable power estimates (e.g., ∼21% in neuroscience; Button et al., 2013; ∼35% in psychological science; Bakker et al., 2012). Based on these findings, improving power in primary studies seems to be desirable in any of the subdisciplines that have been investigated so far and most likely in other disciplines that have not yet been examined.

Third, the examination of effect size trajectories in our regression analyses indicated annual effect size changes of about *r* = 0.01. This means that compared to the initial effect, effect sizes may be expected to shift by a small effect per decade (regardless of the direction of the shift). Consistent with our findings for crude differences, effect declines were more prevalent and stronger than effect increases.

One reason for continuous effect declines in contrast to mere misestimations of the initial study only (i.e., the so-called winner’s curse; Zöllner and Pritchard, 2007) may be due to the anchoring effect of initially published findings for subsequent replications: Suppose an initial research paper reported a moderate (inflated) bivariate effect of *r* = 0.30 although the true effect was *r* = 0.19 (i.e., representing a typical individual differences research effect size, Gignac and Szodorai, 2016). A diligent replicator of such an effect would conduct an *a priori* power analysis and determine a necessary sample size of *n* = 85 to directly replicate the reported effect, thus falling 129 participants short of the necessary 214 participants (i.e., assuming that she used the published effect size for her sample size calculation). With such a number of participants, significance of a hypothesis-conforming effect can only be reached when an extreme (inflated) result is observed.

Therefore, early replications may once more be expected to report inflated effects whilst non-replications (at least in terms of null hypothesis significance testing) are file-drawered (i.e., not published). However, replication sample sizes will eventually increase due to various reasons (e.g., in conceptual replications that require larger samples to detect other effects of interest), thus increasing the probability of obtaining more accurate effect estimates. The observed pattern of lower average power of studies that showed proteus and decline effects than those that showed effect increases is indicative of this phenomenon.

Lower effect size estimates from published successful replications then serve in turn as anchors for further replications, meaning that study power is bound to increase over time. This is consistent with our present findings that the average sample size of primary studies within any given meta-analysis was *n* = 612 (*Md* = 299) which contrasts the low initial study *n*. Therefore, as studies accumulate, the publication of larger-*n* studies will lead to an asymptotical meta-analytical approaching of the true effect. In the long term, this means that even if initial evidence is biased, the true effect will eventually be reached, as time goes by. In the short and medium term, however, biased initial evidence may influence a field for years, entailing an unnecessary strain on resources of unsuccessful replicators and – more importantly – representing an inappropriate effect that is often taken at face value.

We found that based on crude differences, effect inflation in initial studies seemed to be associated with the 2017 impact factor of the journal that the study had been published in. This finding is consistent with the idea that striking findings (which are often difficult to replicate) may be published in higher-profile outlets (Ioannidis, 2005). The detrimental consequences of severe initial effect bias are exacerbated by the observation that initial studies are cited more often than direct or conceptual replications, thus creating unfounded authority (Greenberg, 2009; Voracek, 2014). It seems somewhat reassuring though that annual citation numbers of initial studies were negatively related to effect misestimations in our meta-meta-analysis. Interestingly, we observed no consistent association between initial study publication years and effect misestimations. Considering the comparatively large range of these years (i.e., initial study years ranged from 1913 to 2005), this may mean that publication pressure plays only a limited role in driving effect biasing mechanisms. However, this idea warrants further systematic investigation of larger meta-meta-analytic datasets to allow a reliable interpretation.

The interpretation of decline effects as functions of low (initial) study power and precision are largely supported by our moderator analyses: Negative associations of initial study sample sizes with effect misestimations in both crude and regression slope-based analyses yielded larger effect sizes for declines than for overall time trends. Moreover, the average power of primary studies was considerably larger for studies belonging to increasing than for those belonging to declining or proteus effects. These differences in study power were even more pronounced when only initial studies were included. Summary effect strengths were predominantly negatively associated with overall and declining effect sizes. This result is consistent with our finding of lower initial study power (i.e., representing smaller sample sizes and study precision) being associated with stronger effect declines because larger true effects can be more accurately (i.e., less biased) detected in designs with smaller sample sizes. Consequently, underpowered studies that only reach significance when observing inflated effects seem to drive effect misestimations. Our findings, that moderating effects of initial study properties and the strength of the meta-analytical summary effects yielded typically stronger effects on declines than on effect changes on the whole supports the idea that these moderators are potent drivers of declines, but not of all cross-temporal changes.

The findings of our meta-meta-analysis have important implications for science policy and stress the necessity of adopting more rigorous approaches when it comes to designing, conducting, reporting, and eventually interpreting the findings of exploratory and replication studies. Preregistering (exploratory) studies in time-stamped online resources such as the Open Science Framework^1^ and publishing the primary data may be a good start (for a discussion, see Nuijten et al., 2018). However, it seems worthwhile to encourage exploratory authors to replicate their findings prior to publication (i.e., by themselves or by facilitating pre-publication independent direct replication; see, Tierney et al., 2018). Although direct self-replication may, due to practical reasons, not be possible in all cases and will most likely not be able to prevent all occurrences of bias, discovery-replication sampling approaches have proved their worth in neuroscience (e.g., Kang et al., 2015) and are likely to be just as useful for exploratory research in other empirical disciplines.

Pre-publication direct self- and independent replications may help in making initial effect estimates more reliable and subsequent studies more adequately powered. Without (self-)replication the face-value interpretation of exploratory effects remains dubious. Therefore, potential replicators of exploratory studies may wish to use concepts such as safeguard power (i.e., anchoring their power analysis on the lower threshold of the confidence interval instead of on the effect estimate; see Perugini et al., 2014) to design direct or conceptual replications.

### Limitations

First, it needs to be acknowledged, that contrary to our expectations, our dissemination bias analyses showed about equal (or even numerically smaller) numbers of publication bias indications in meta-analyses that were associated with effect declines and those associated with increases. However, publication bias is typically assumed to be mainly driven by mere significance of results and bias susceptibility may therefore not depend on phenomena that are related to cross-temporal declines or increases. Of note, in our publication bias analyses all primary studies that had been included within the meta-analyses have been treated as if they had been published which necessarily leads to an underestimation of the publication bias prevalence. Therefore, our estimate must be seen as a lower threshold of actual publication bias occurrence.

Second, signs of predictors in our moderator analyses were not unequivocally consistent across different calculation methods. Although we focused on interpreting the most sophisticated estimate throughout our paper (i.e., weighted mixed-effects estimations), we provided fixed-effect and unweighted estimates in our Table 2 to allow readers to evaluate our results based on different methods of effect estimation.

In this vein, it should be noted that moderator analyses appeared to have stronger and more consistent effects in analyses of crude differences than of regression coefficients. This is unsurprising, because less potential confounding variables (e.g., number of primary studies within meta-analyses; within study-year variance of data points) are involved in the calculation of crude differences. Moreover, future researchers may wish to investigate how primary study characteristics within individual meta-analyses such as certain design features relate to the decline effect.

Third, using the first published study effect as a reference point in our crude analyses may have introduced some noise in our data in those cases where identical exploratory hypotheses within individual meta-analyses were coincidentally investigated and published in two independent studies around the same time (i.e., yielding initial and subsequent publication(s) that are only a few months apart). However, this should have only played a minor role in our analyses, because initial studies had unique publication years in all meta-analyses excepting two (in one further individual case, the initial effect size was calculated as a weighted average of two independent effects, because the publication order within the first publication year could not be established).

Fourth, we used 2017 instead of initial study publication year impact factors in our analyses because most initial study publication years predate the introduction of this metric in 1997. However, using current impact factors may be considered to be reasonable proxies for the relative reputation of the journals that initial studies have been published in, although for these calculations some additional noise may be expected in our data.

Finally, we acknowledge that our data were limited to meta-analyses from a specialized journal in a specific academic subfield and may therefore not be generalizable to the entire psychological literature. However, the observed power of the primary studies that the included meta-analyses comprised exceeded estimates from other psychological subdisciplines considerably (e.g., median power in neuroscience ∼ 8% to 31%; Button et al., 2013; mean power in psychology ∼ 35%; Bakker et al., 2012) averaging around (still suboptimal) 48% to 50%. Because low study power must be considered to represent a major driver of decline effects, it seems likely that the present observations are not confined to papers that have been published in *Intelligence* and may be observed in a conceivably even more pronounced fashion in other subdisciplines.

### Final Words

We show in the present meta-meta-analysis evidence for overproportional (at a ratio of 2:1) and stronger effect declines than increases in the published intelligence literature. Effect misestimations are most likely due to low initial study power and strategic research and submission behaviors of exploratory researchers and replicators alike. These misestimations are non-trivial in nature and correspond to a change of a small effect size per decade (*r*∼0.10) in either direction compared to the initial effect.

Considering the diversity of research questions within the included meta-analyses and the comparatively high power of primary studies, it seems likely that the present results may not be confined to intelligence research, but may be expected to generalize to psychological science and perhaps empirical sciences in general. Extensive implementations of study preregistration, publication of primary data, discovery-(direct)replication sampling approaches, and the use of safeguard power in replications may help in alleviating problems that are associated with effect misestimation.

## Data Availability Statement

All data are provided in the online Supplementary Data Sheet S3; the R-Code can be found in online Supplementary Data Sheet S4.

## Author Contributions

JP designed the study, piloted coding sheets, coded and obtained the full texts, and wrote the draft. JP and MS performed the analyses and interpreted the data. MS contributed toward coding by resolving inconsistencies in coding when necessary. All authors contributed to critically revising and amending the manuscript drafts.

## Conflict of Interest

The authors declare that the research was conducted in the absence of any commercial or financial relationships that could be construed as a potential conflict of interest.

## Footnotes

1 www.osf.io
