# Supplementary material for: Effect Declines Are Systematic, Strong, and Ubiquitous: A Meta-Meta-Analysis of the Decline Effect in Intelligence Research
Source: Front Psychol. 2019 Dec 19;10:2874. doi: 10.3389/fpsyg.2019.02874 (PMC6930891; doi:10.3389/fpsyg.2019.02874)

Baker et al. (2014)

Initial study number: 4

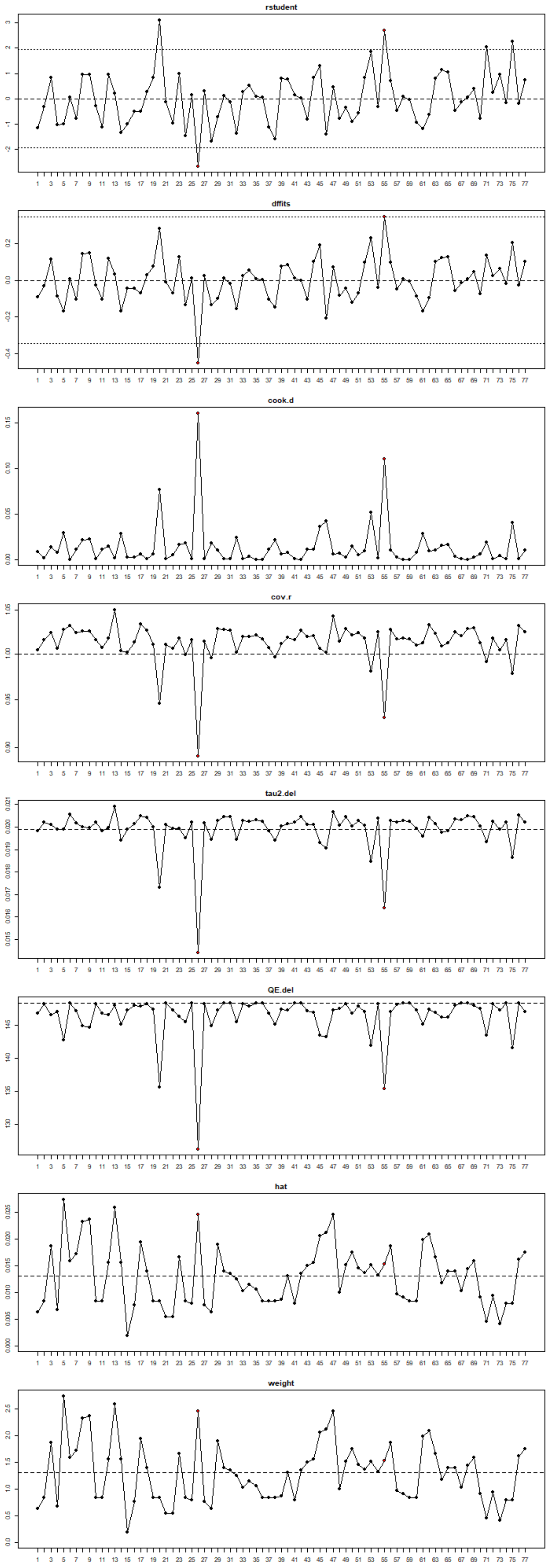

Banks et al (2010)

Initial study number: 4

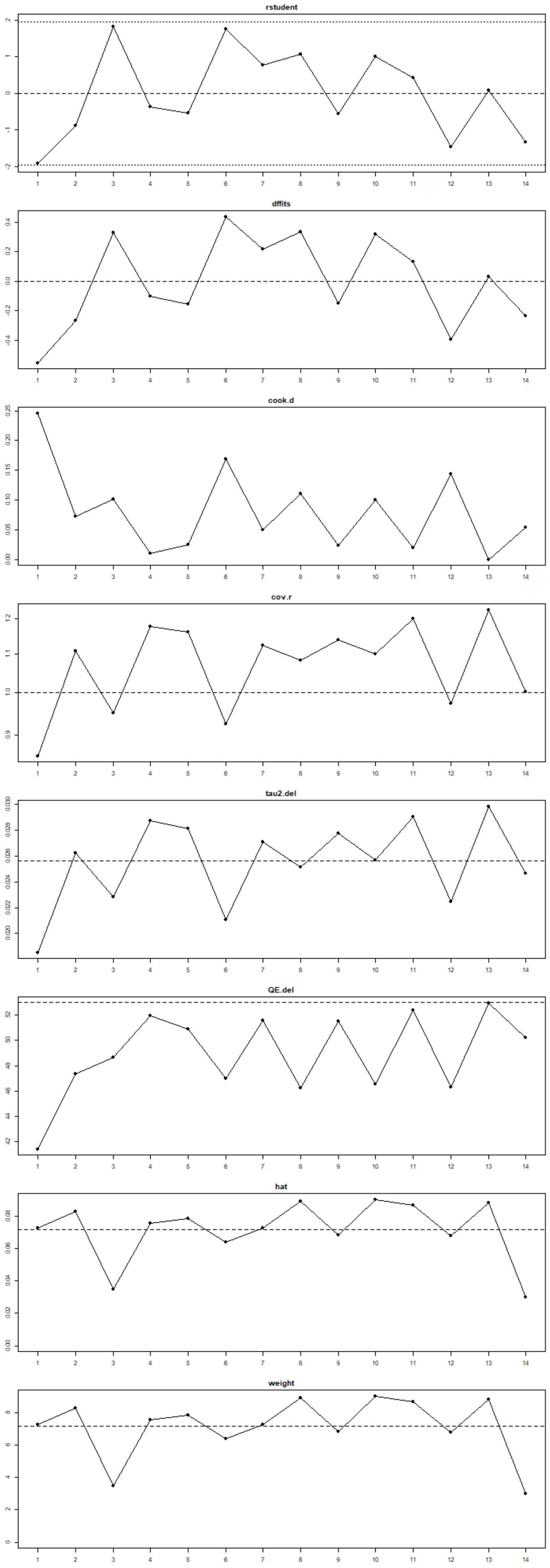

Beaujean (2005)

Initial study: 1

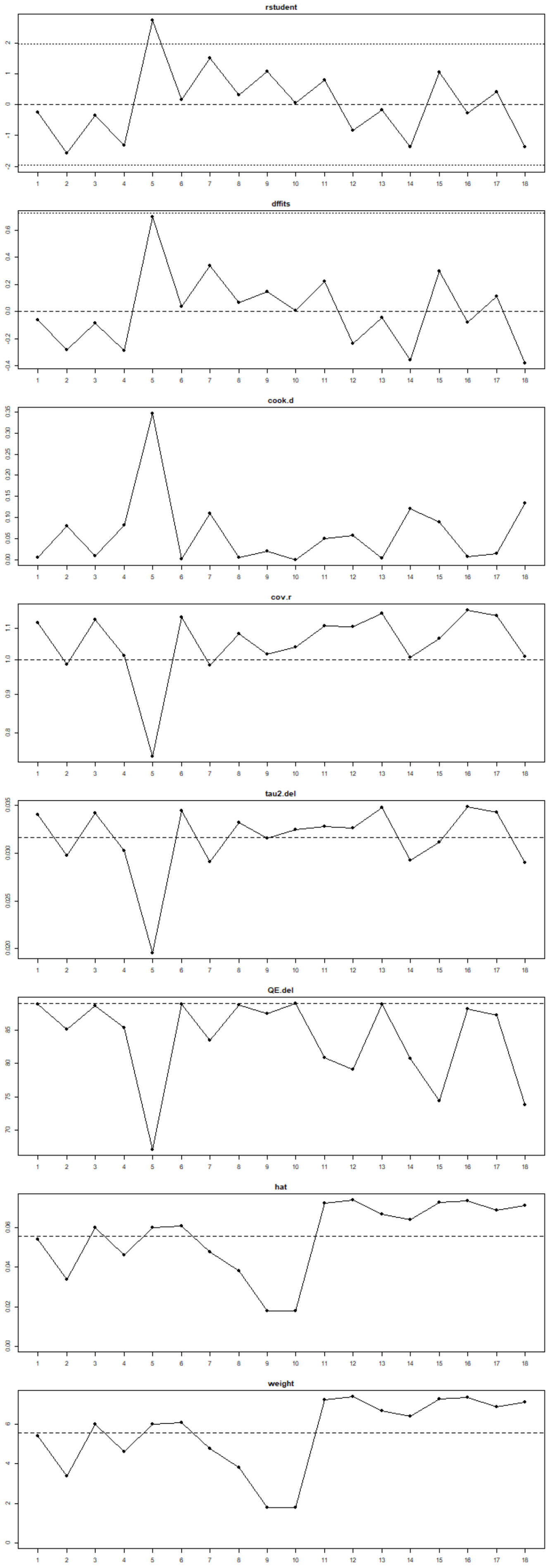

Geller et al (2017) (1)

Initial study: 7

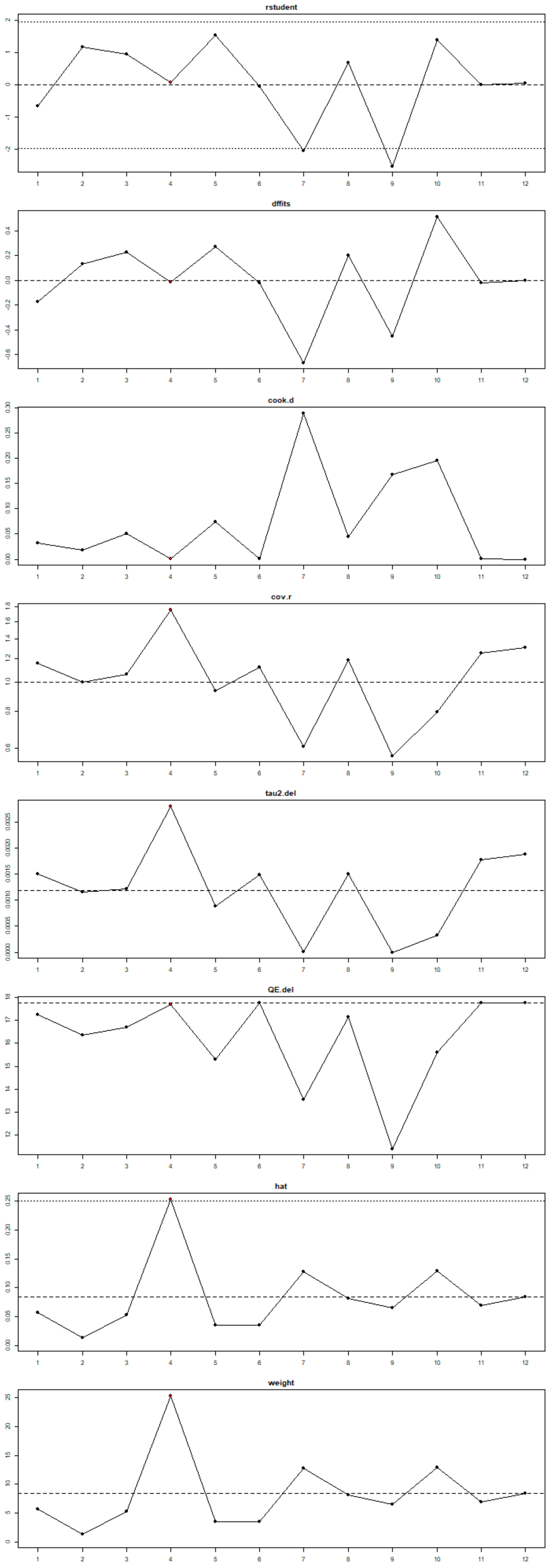

Geller et al (2017) (2)

Initial study: 1

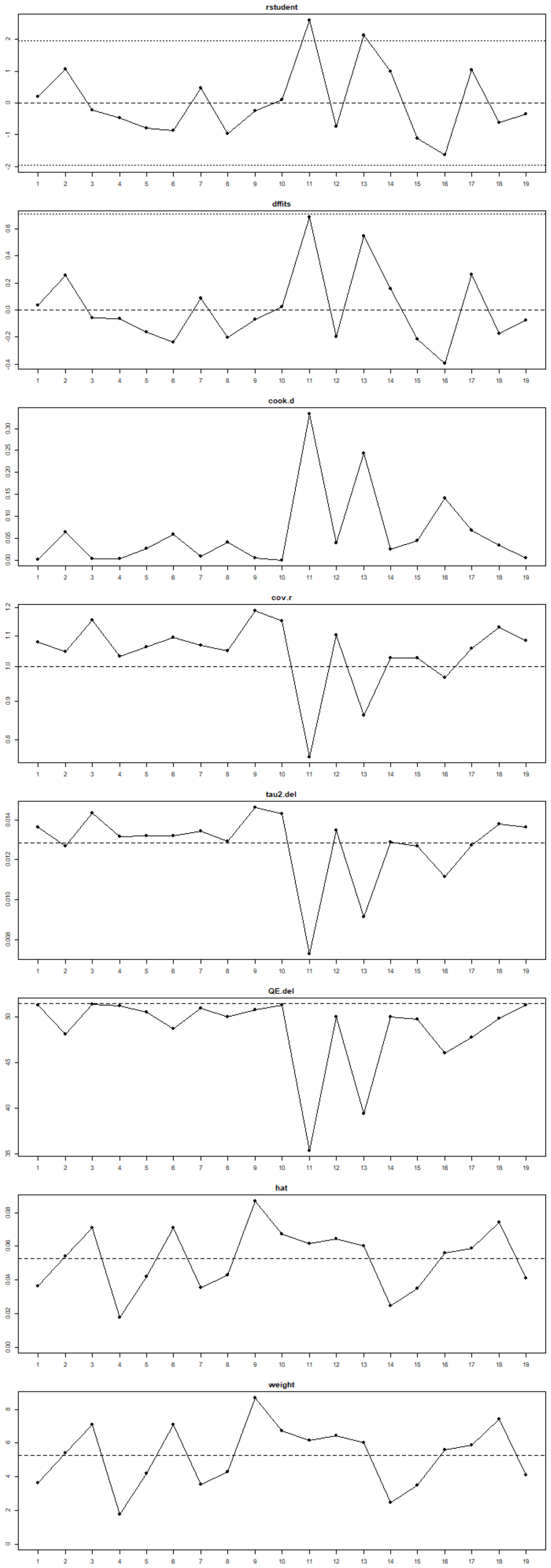

Gignac & Bates (2017)

Initial study: 1

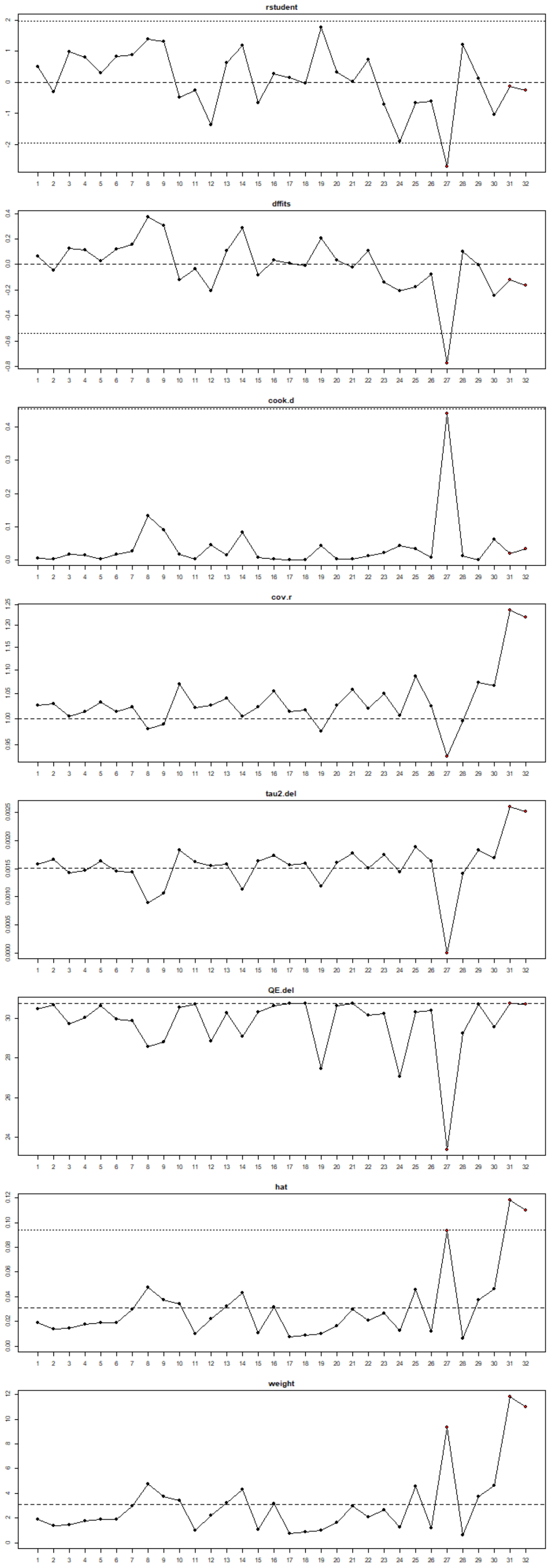

Grudnik & Kranzler (2001)

Initial study: 1

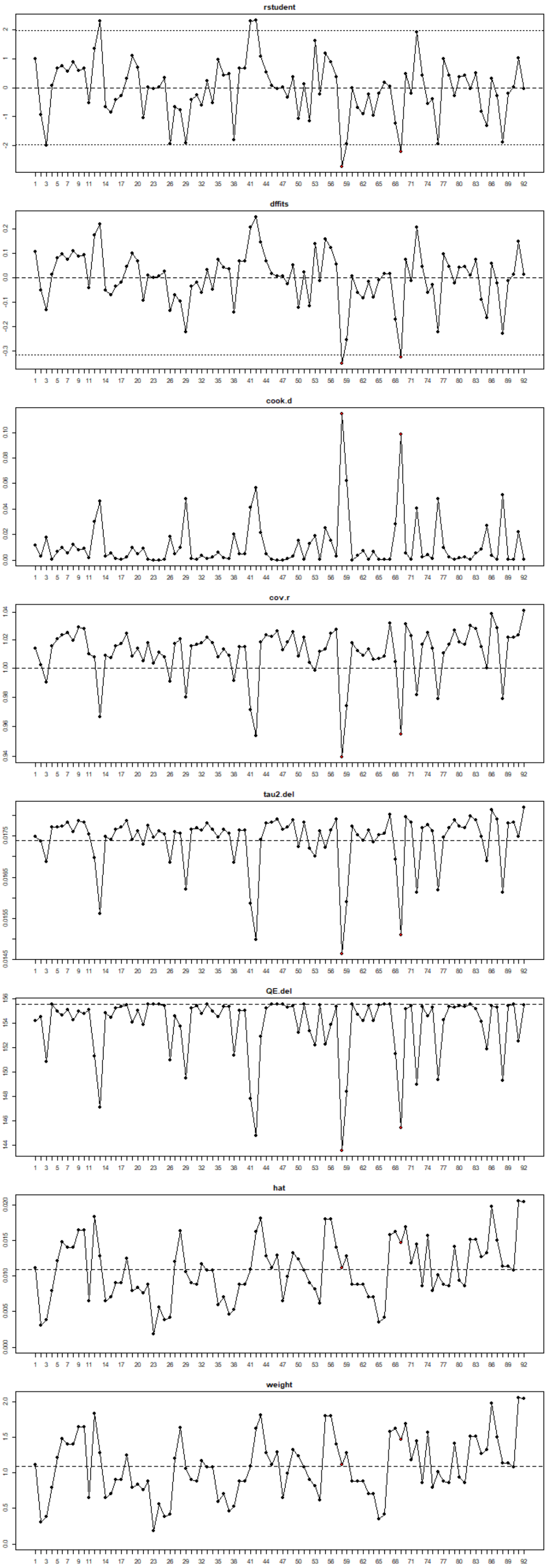

Kranzler & Jensen (1989)

Initial study: 19

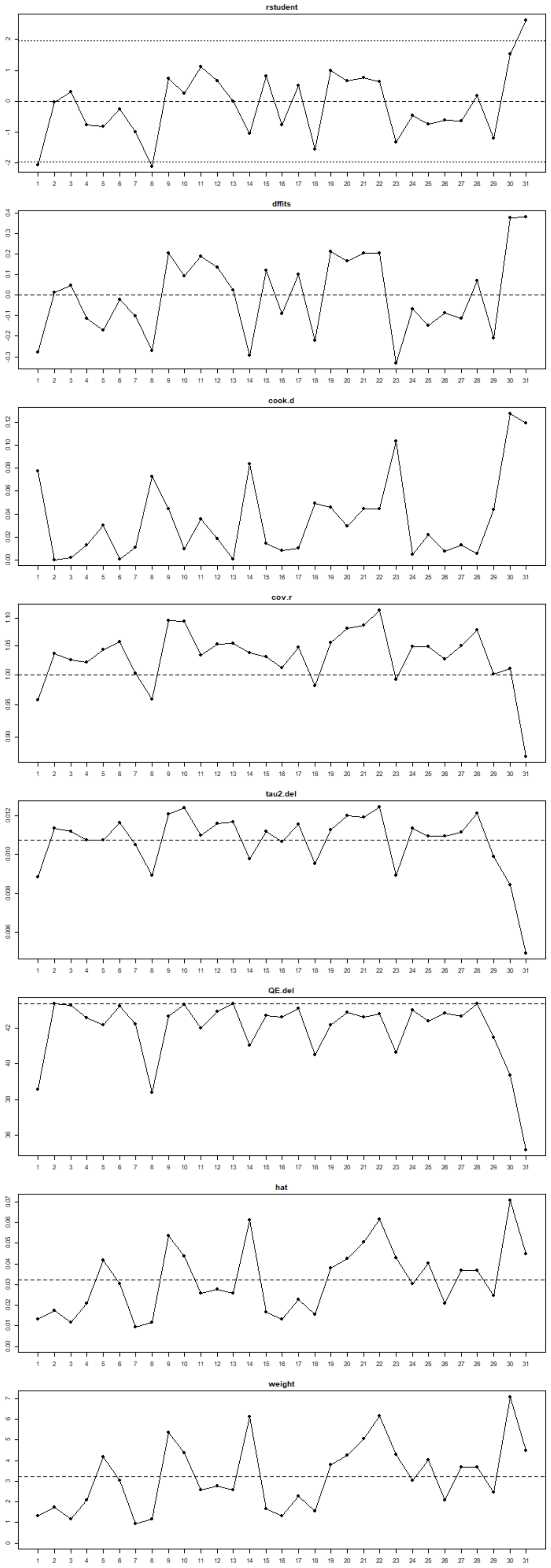

Lynn & Irwing 1(2008)

Initial study: 5

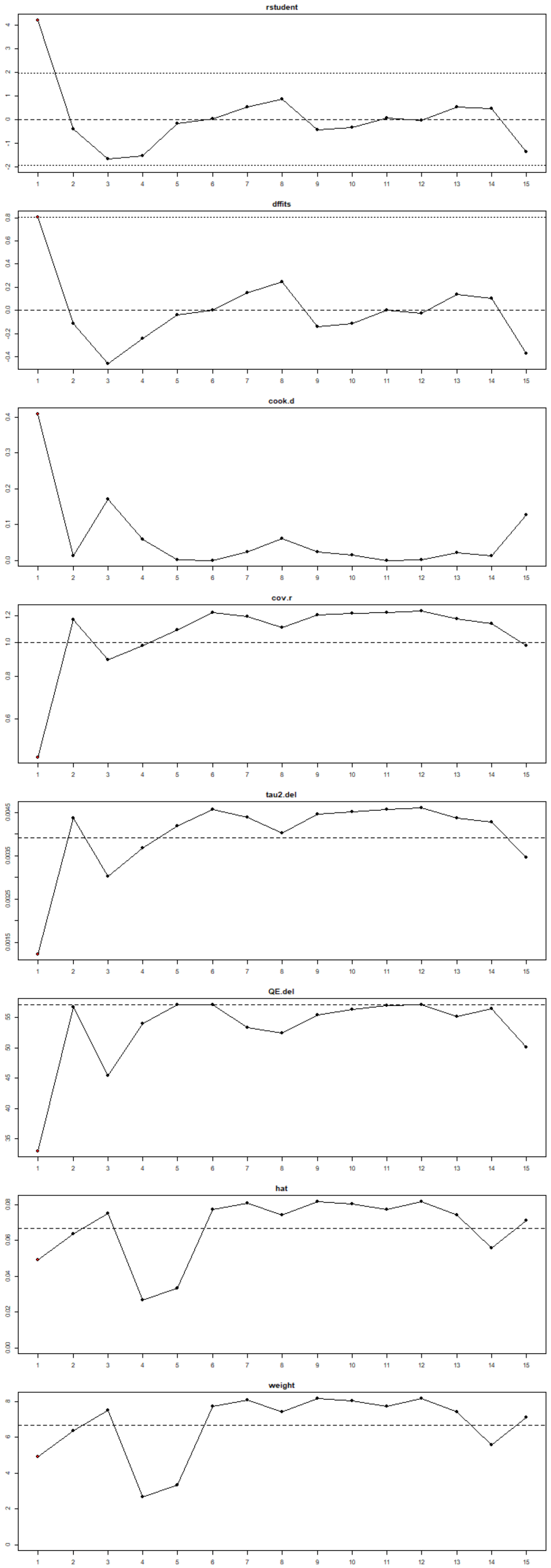

Lynn & Irwing 2(2008)

Initial study: 1

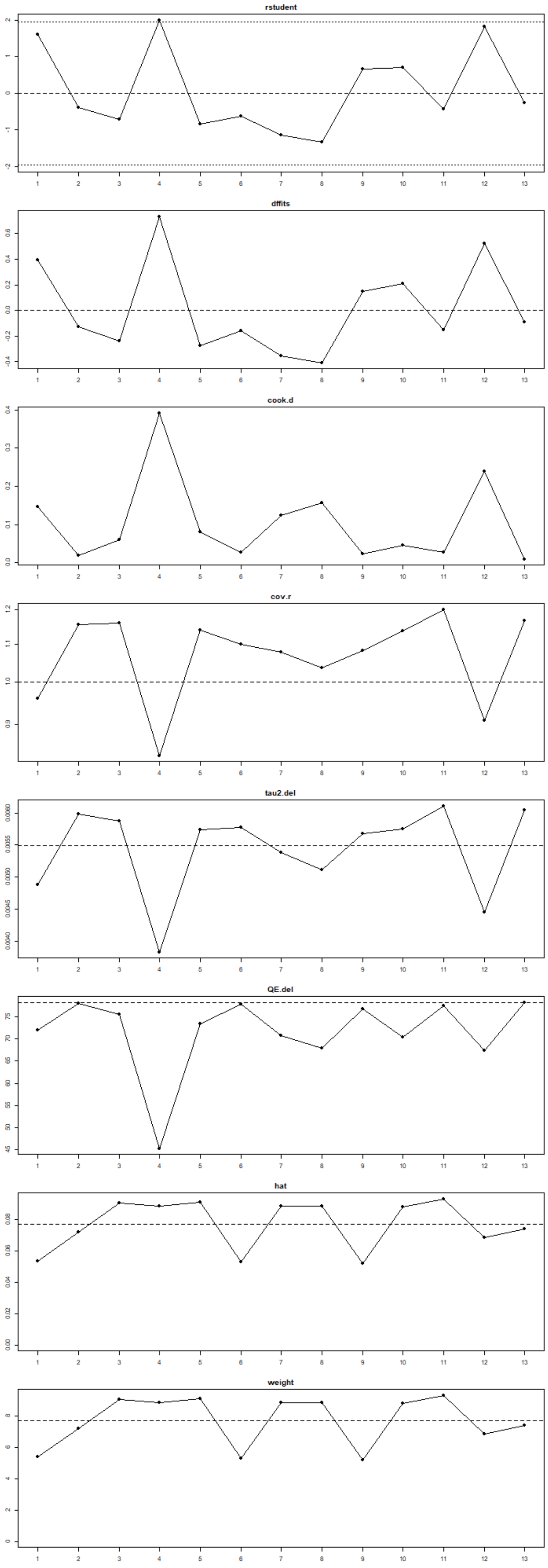

Lynn & Irwing 3(2008)

Initial study: 1

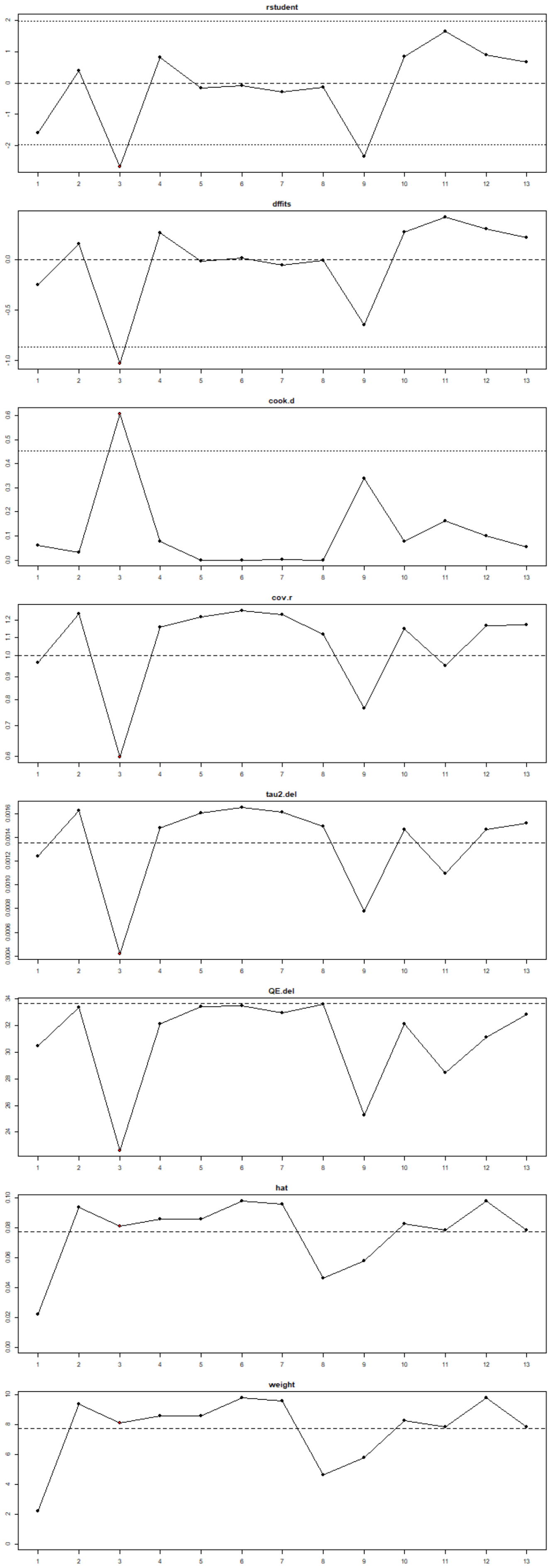

Lynn & Irwing 4(2008)

Initial study: 1

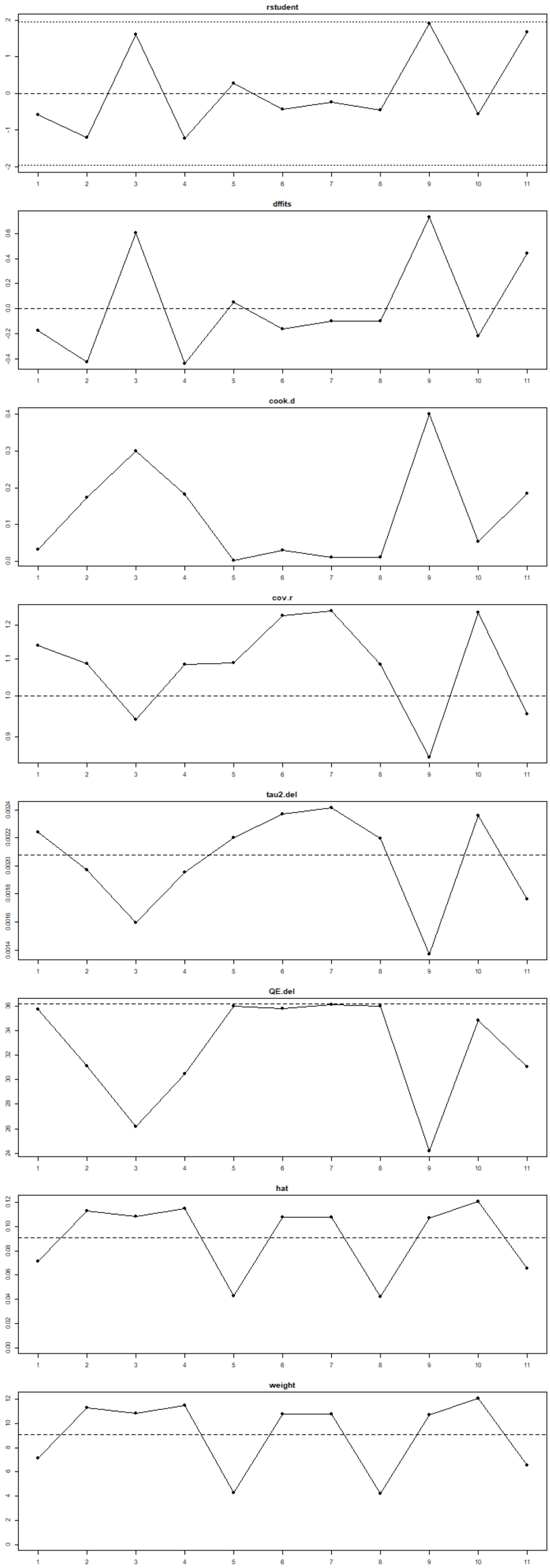

McDaniel (2005)

Initial study: 1

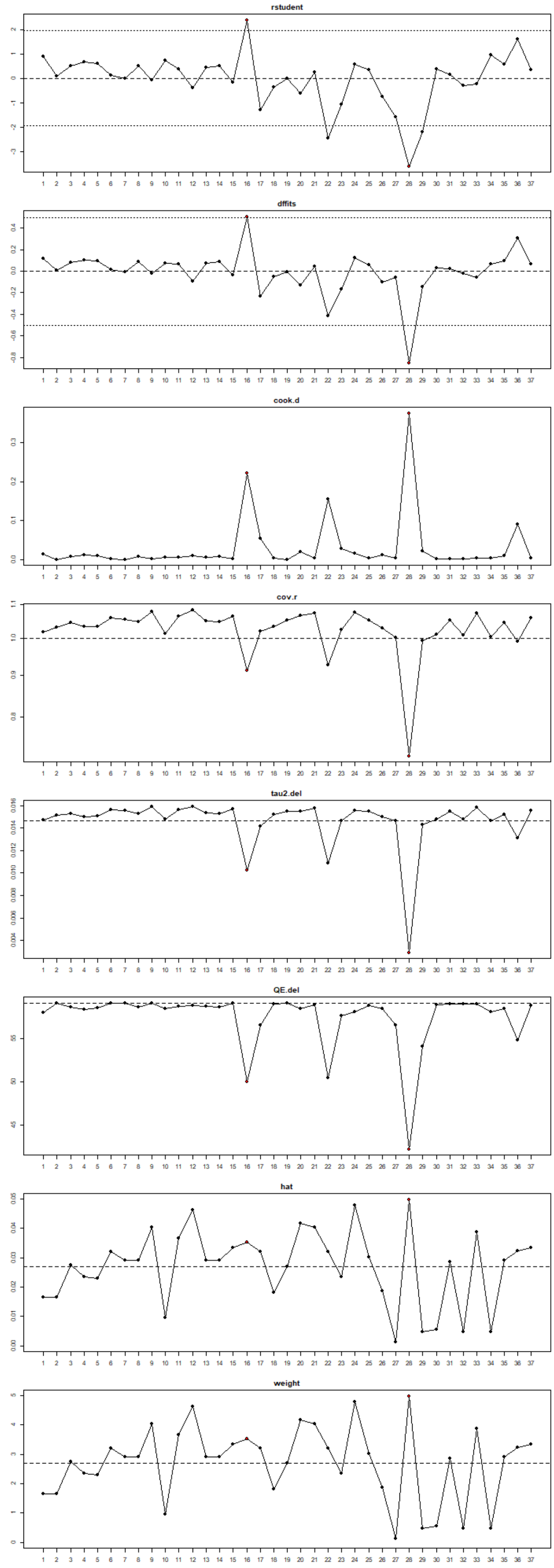

Murphy & Hall (2011)

Initial study: 15

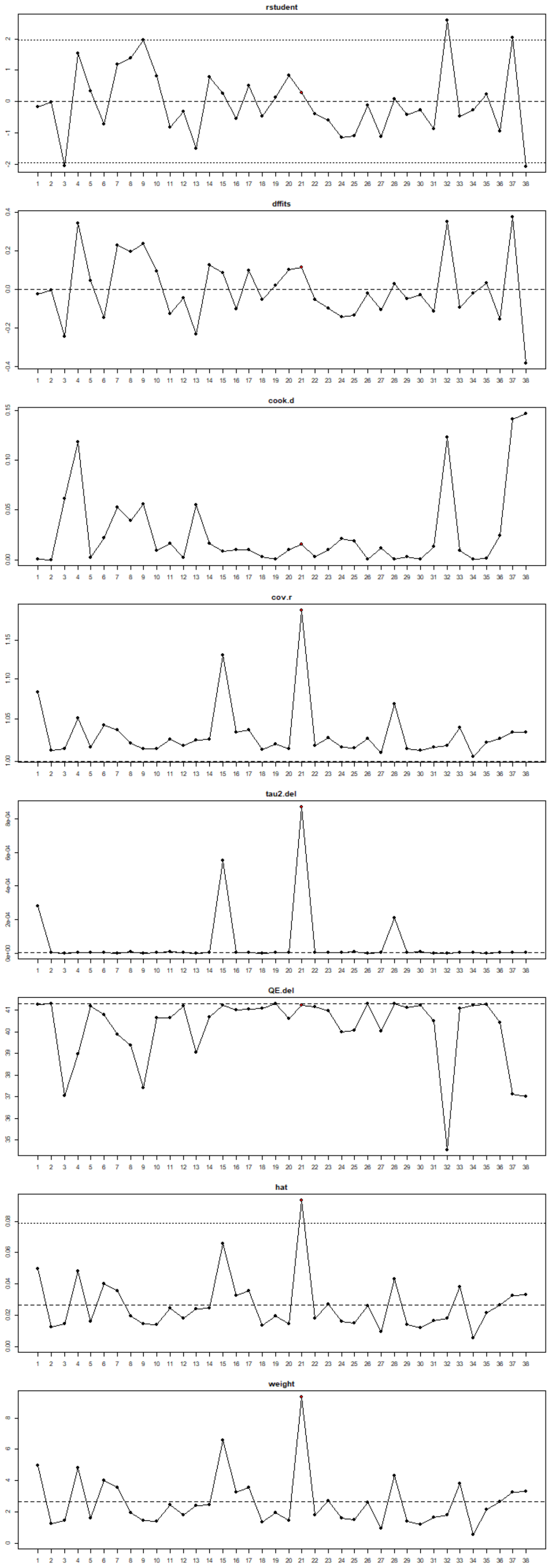

Paessler et al(2015)_A

Initial study: 8

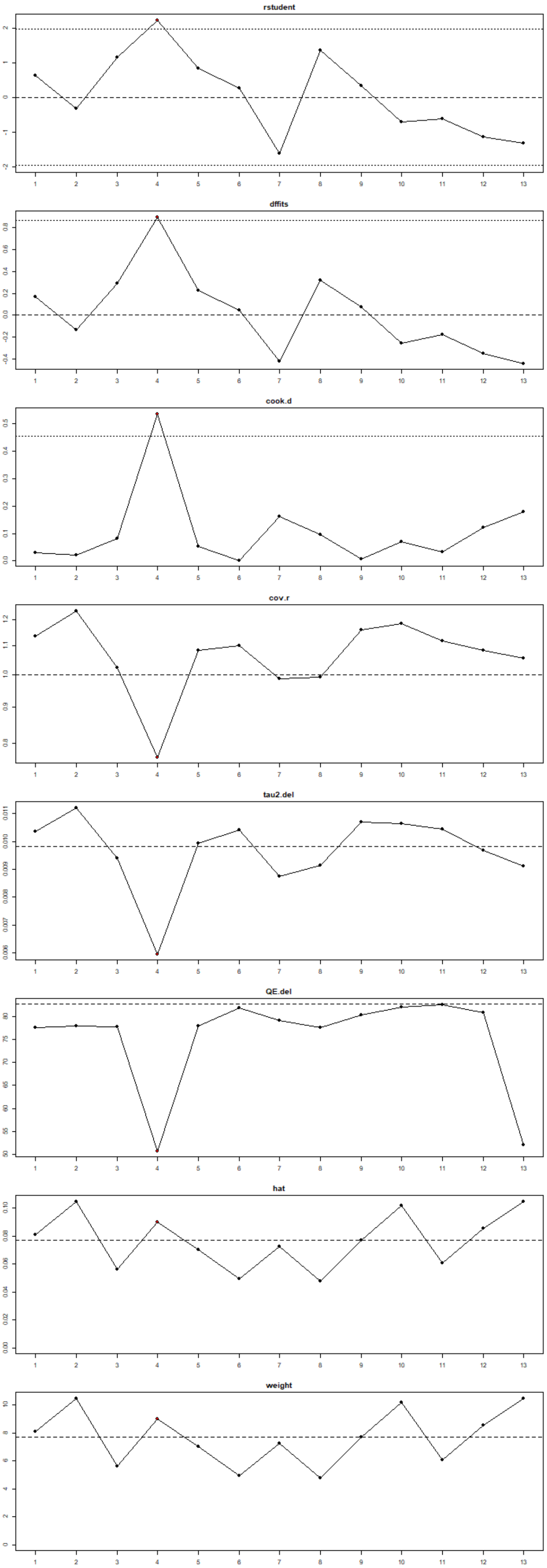

Paessler et al(2015)_C

Initial study: 3

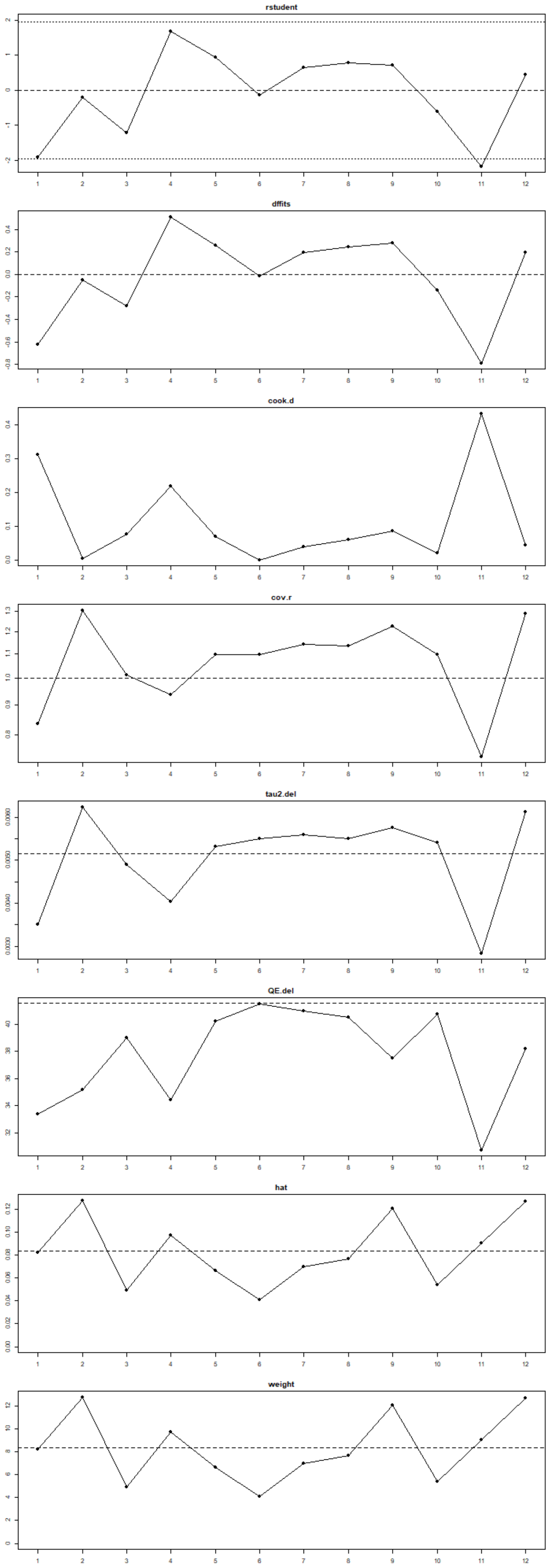

Paessler et al(2015)_E

Initial study: 1

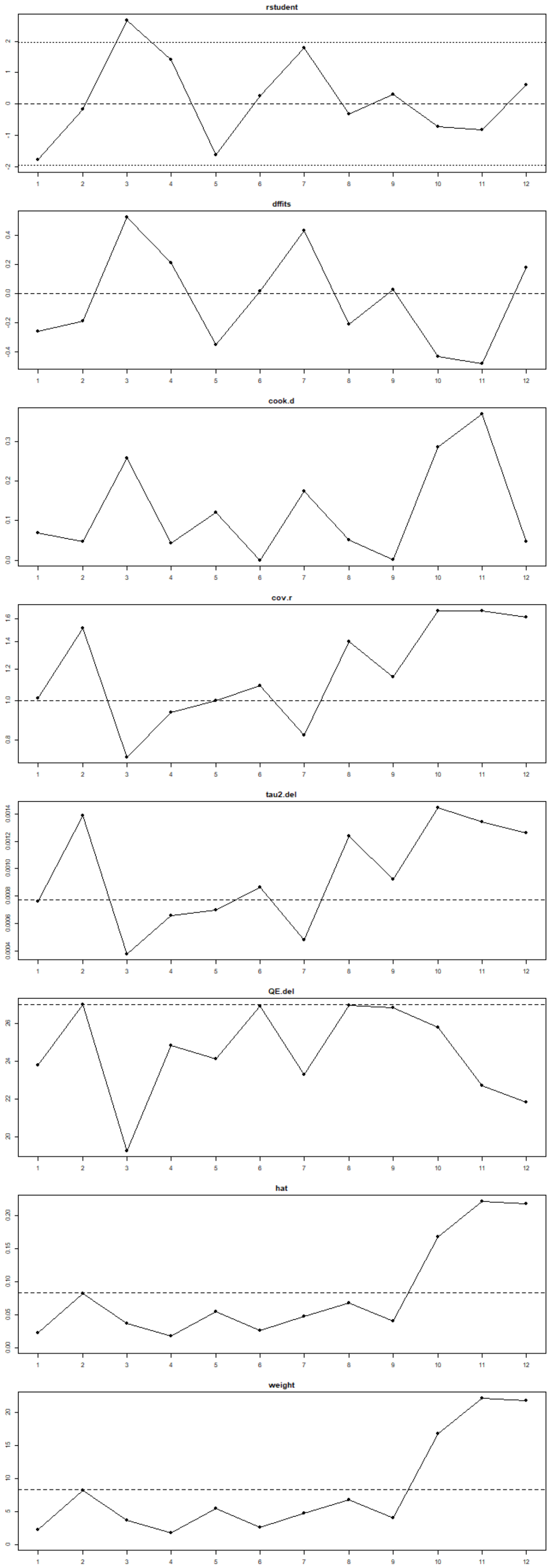

Paessler et al(2015)_I

Initial study: 8

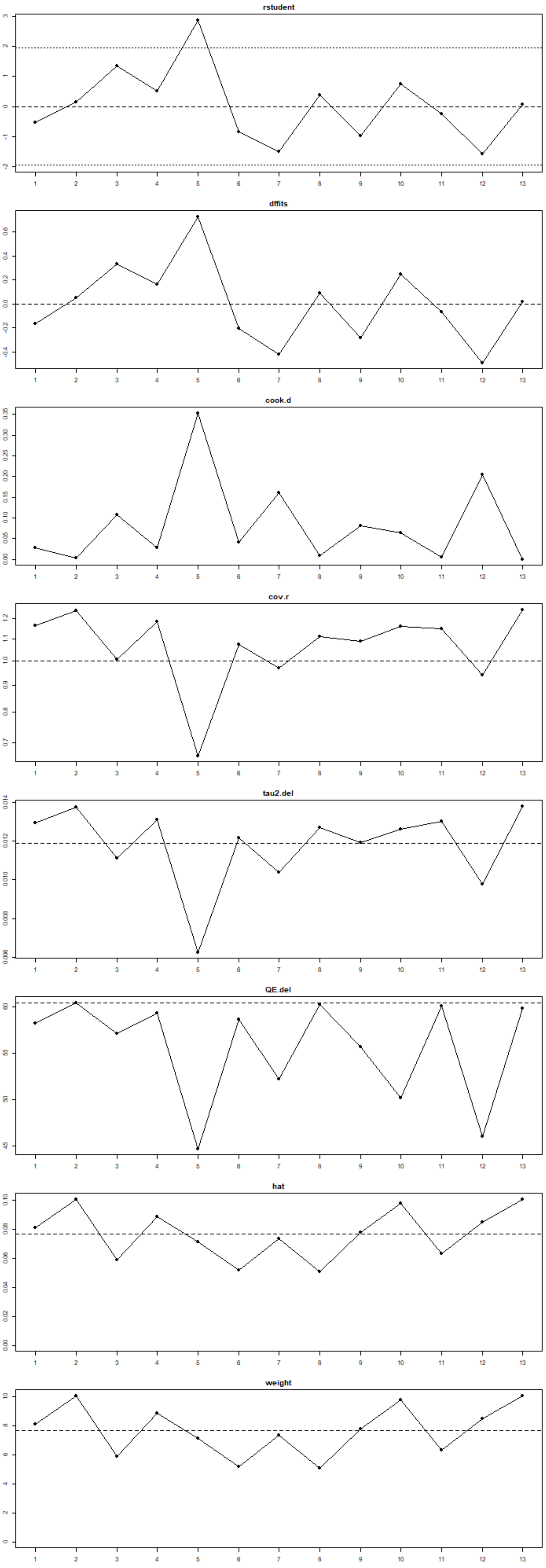

Paessler et al(2015)_R

Initial study: 1

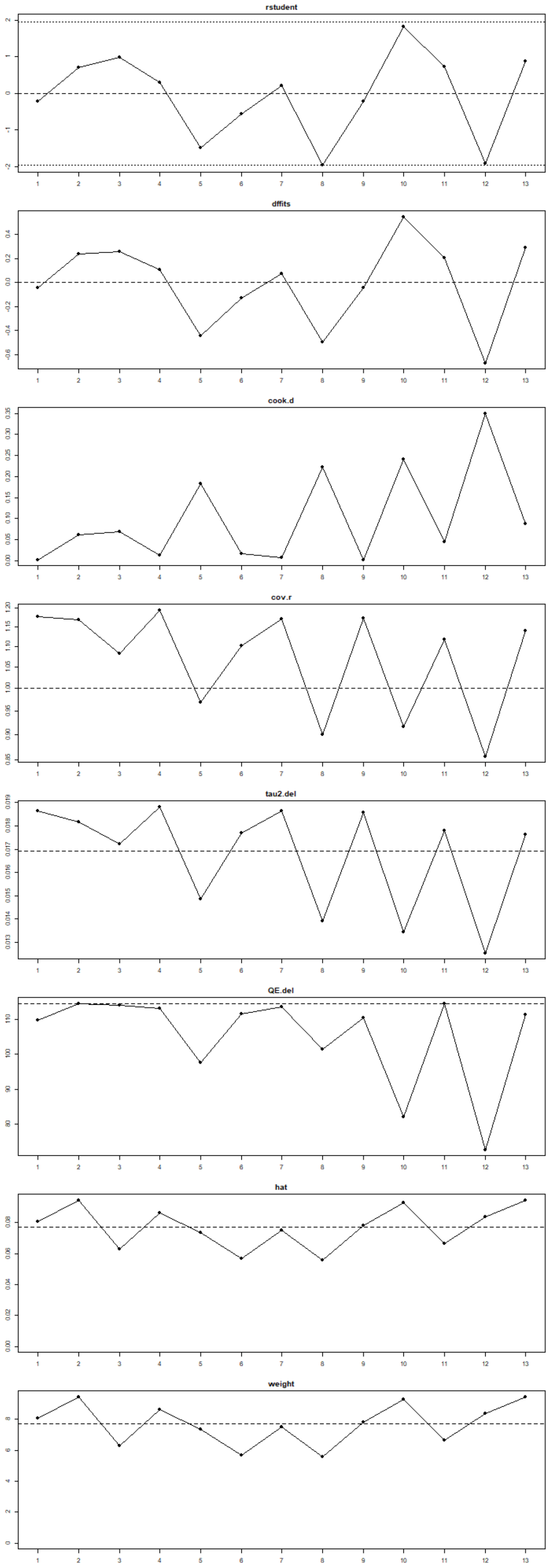

Paessler et al(2015)_S

Initial study: 1

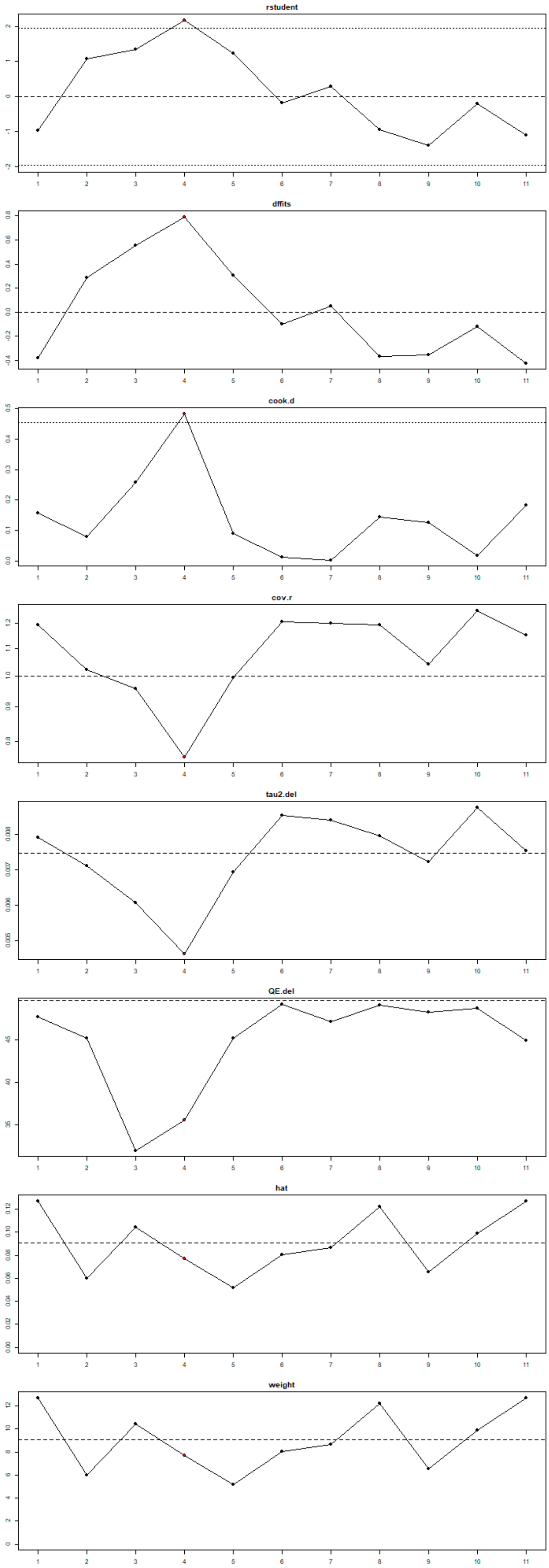

Pietschnig & Gittler (2017)

Initial study: 1

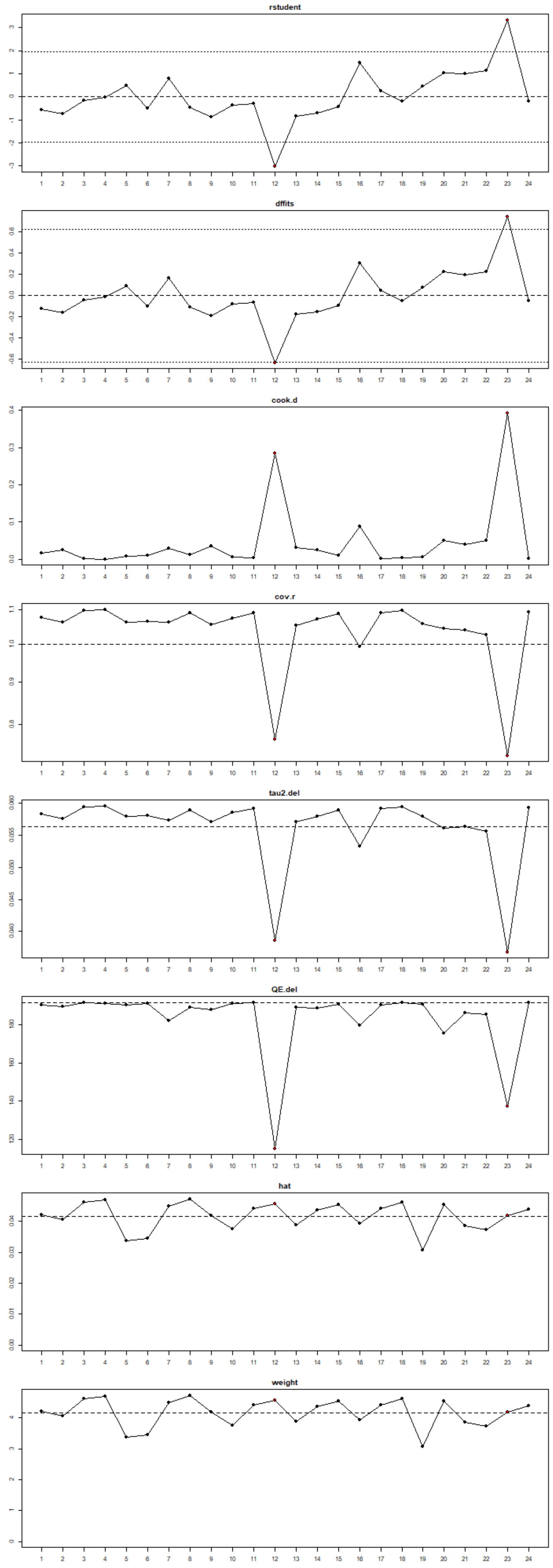

Pietschnig et al. (2010) monm

Initial study: 1

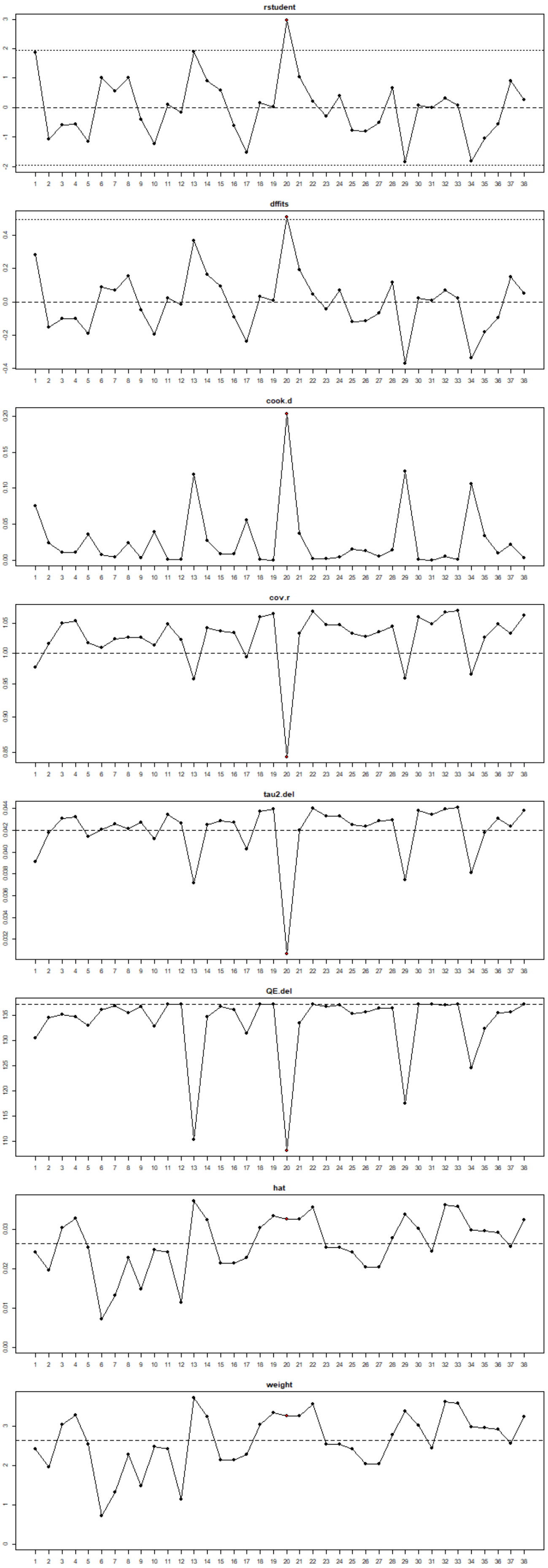

Pietschnig et al. (2010) moom

Initial study: 1

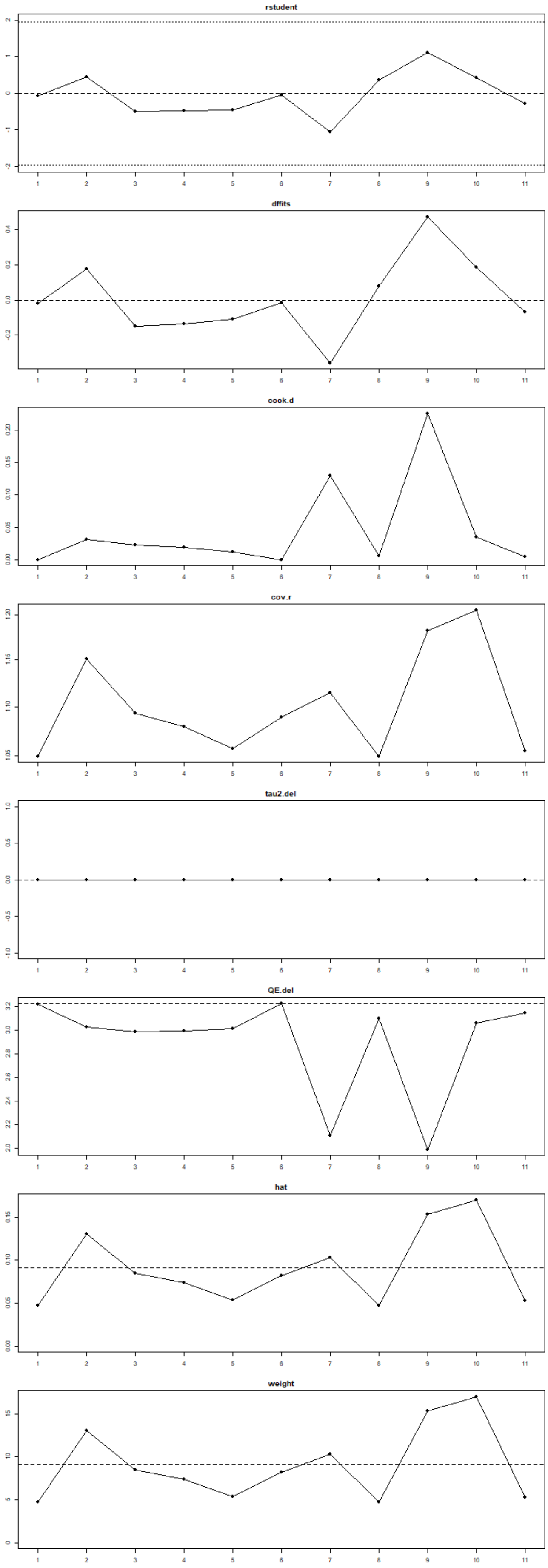

Pietschnig et al. (2010) omnm

Initial study: 1

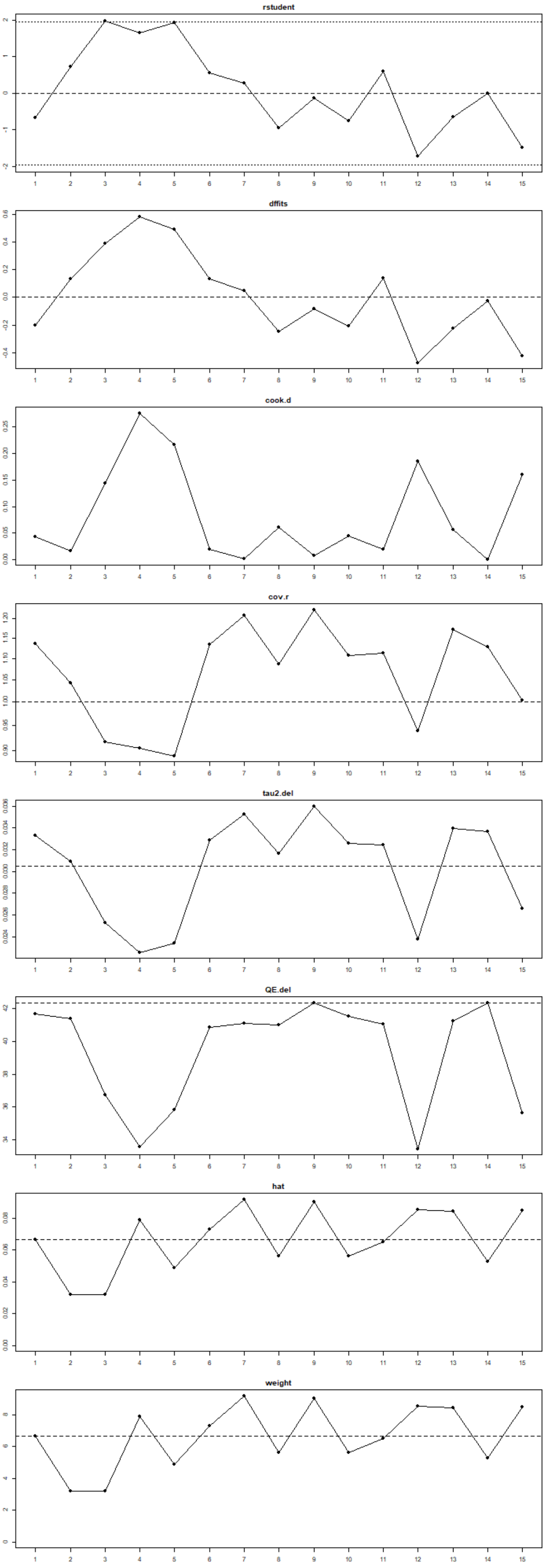

Roth et al (2015)

Initial study: 13

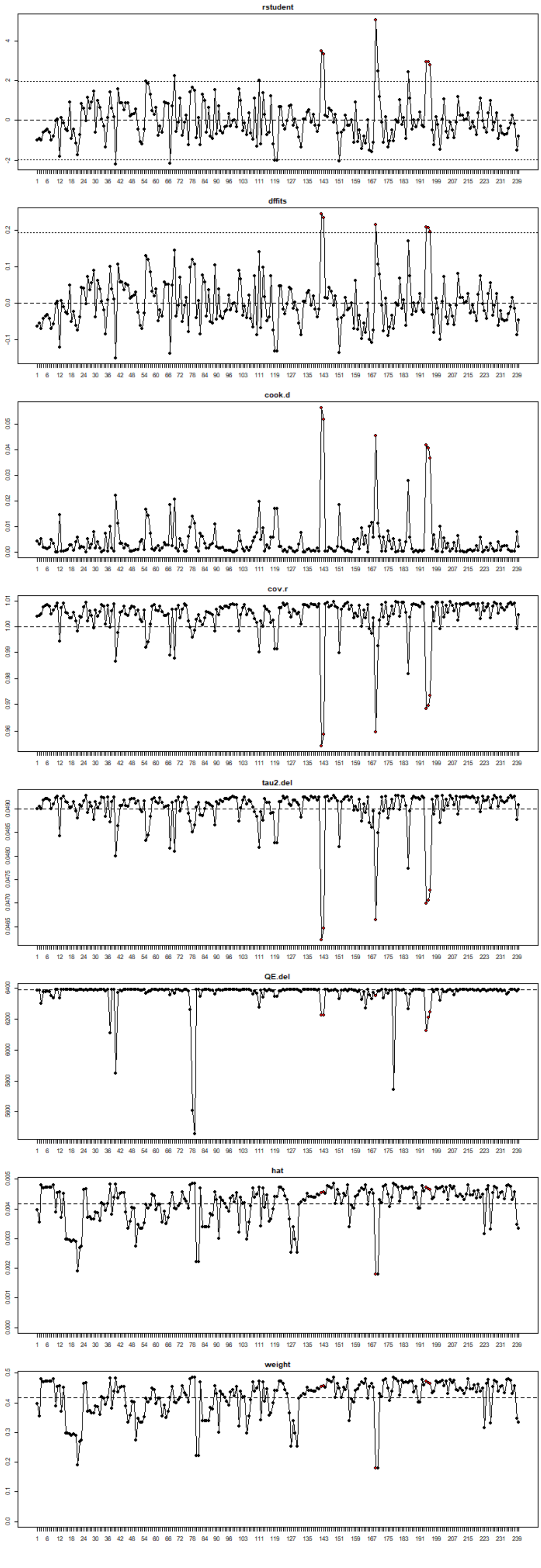

Sala et al (2017)

Initial study: 1

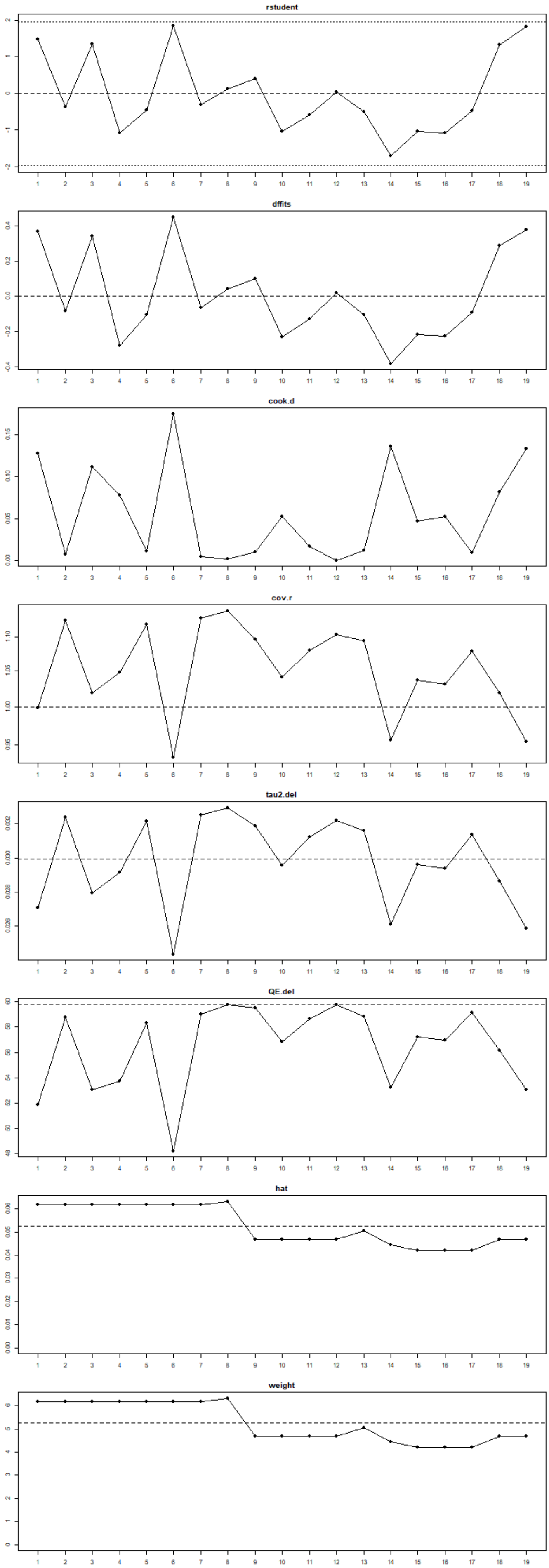

Shamosh & Gray (2008)

Initial study: 1

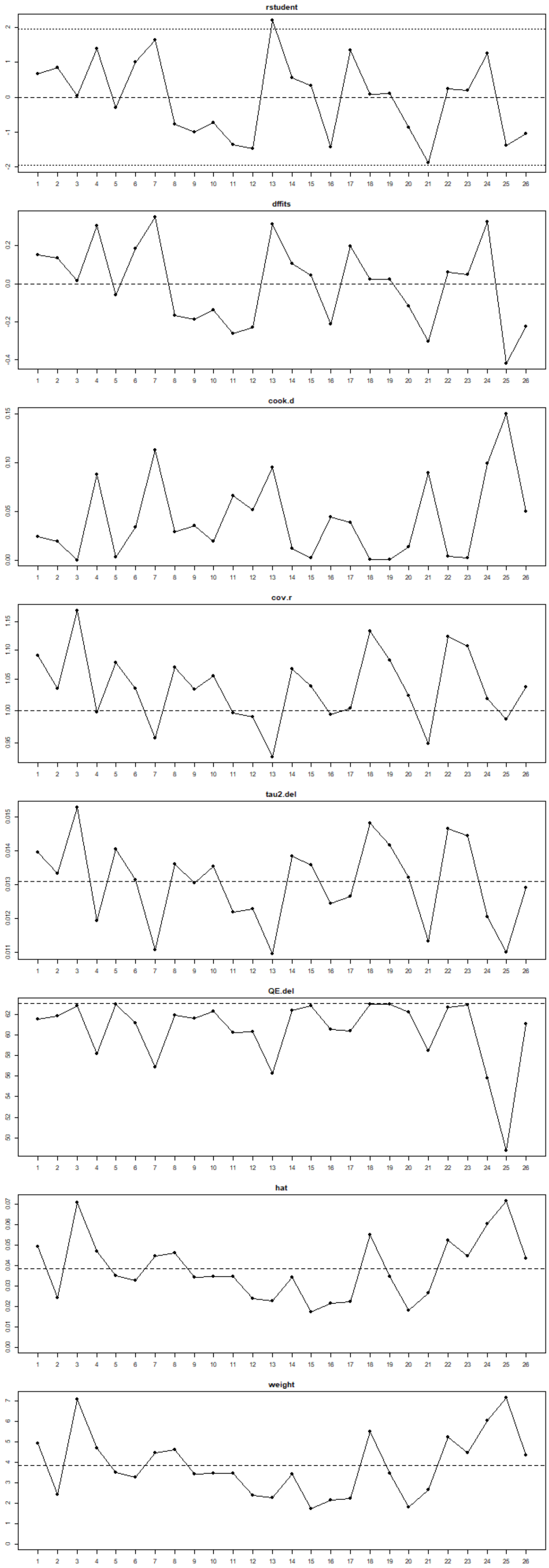

Stadler et al (2015)

Initial study: 1

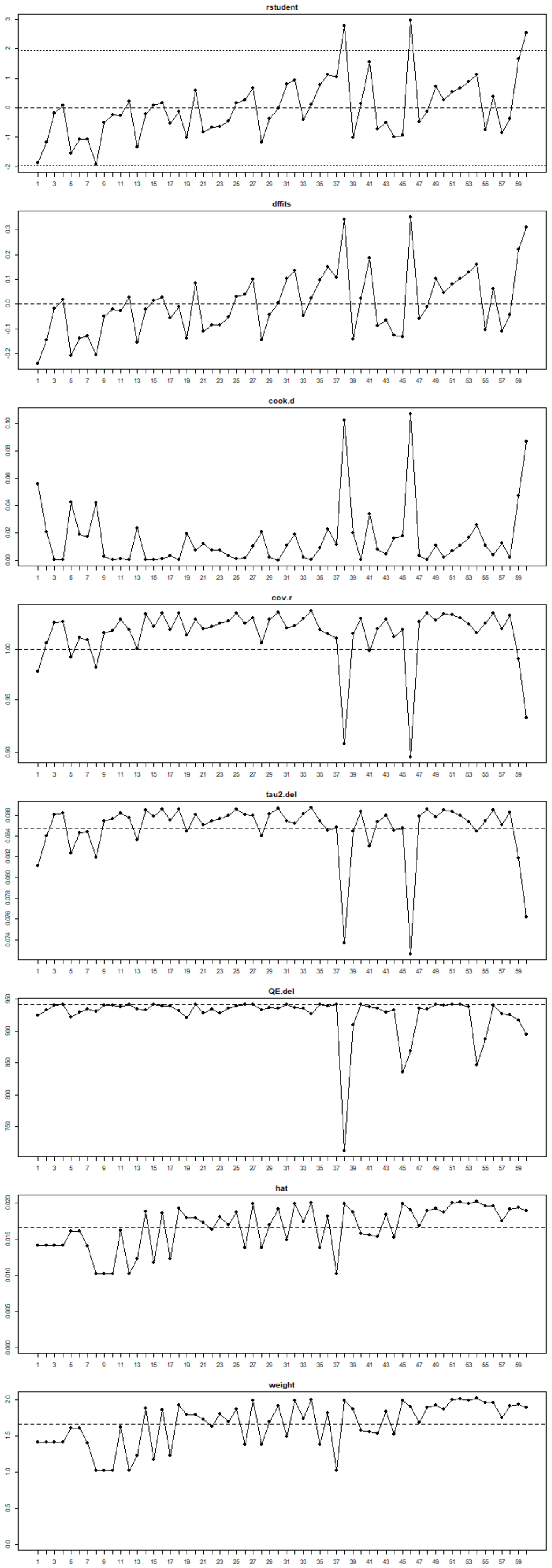

Webster & Duffy (2016)

Initial study: 1

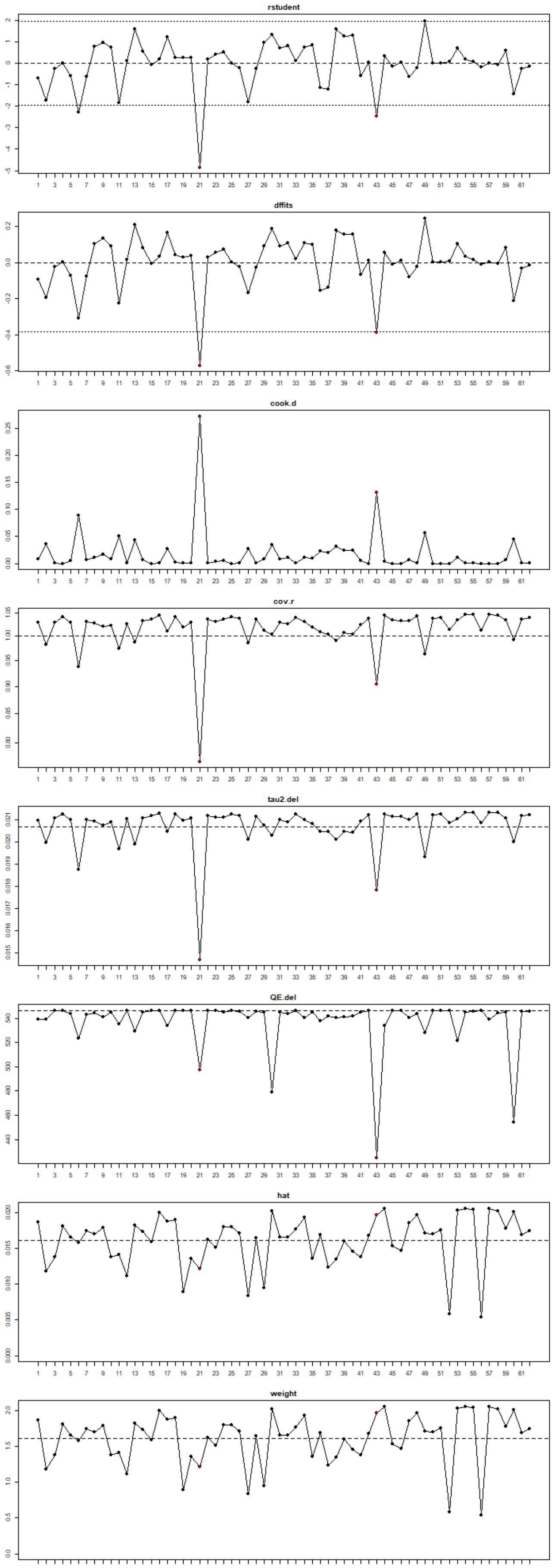

Supplement: Supplementary file 1 [file Data_Sheet_1.pdf]
