# Supplementary material for: Effect Declines Are Systematic, Strong, and Ubiquitous: A Meta-Meta-Analysis of the Decline Effect in Intelligence Research
Source: Front Psychol. 2019 Dec 19;10:2874. doi: 10.3389/fpsyg.2019.02874 (PMC6930891; doi:10.3389/fpsyg.2019.02874)
Supplement: Supplementary file 3 [file Data_Sheet_3.docx]

**Included Meta-Analyses**

Baker, C. A., Peterson, E., Pulos, S., & Kirkland, R. A. (2014). Eyes and IQ: A meta-analysis of the relationship between intelligence and “Reading the Mind in the Eyes.” *Intelligence*, *44*, 78-92. https://doi.org/10.1016/j.intell.2014.03.001

Banks, G. C., Batchelor, J. H., & McDaniel, M. A. (2010). Smarter people are (a bit) more symmetrical: A meta-analysis of the relationship between intelligence and fluctuating asymmetry. *Intelligence*, *38*(4), 393-401. https://doi.org/10.1016/j.intell.2010.04.003

Beaujean, A. A. (2005). Heritability of cognitive abilities as measured by mental chronometric tasks: A meta-analysis. *Intelligence*, *33*(2), 187-201. https://doi.org/10.1016/j.intell.2004.08.001

Geller, S., Wilhelm, O., Wacker, J., Hamm, A., & Hildebrandt, A. (2017). Associations of the COMT Val(158)Met polymorphism with working memory and intelligence - A review and meta-analysis. *Intelligence*, *65*, 75-92. https://doi.org/10.1016/j.intell.2017.09.002

Gignac, G. E., & Bates, T. C. (2017). Brain volume and intelligence: The moderating role of intelligence measurement quality. *Intelligence*, *64*, 18-29. https://doi.org/10.1016/j.intell.2017.06.004

Grudnik, J. L., & Kranzler, J. H. (2001). Meta-analysis of the relationship between intelligence and inspection time. *Intelligence*, *29*(6), 523-535. https://doi.org/10.1016/S0160-2896(01)00078-2

Kranzler, J., & Jensen, A. (1989). Inspection Time and Intelligence - a Meta-Analysis. *Intelligence*, *13*(4), 329-347. https://doi.org/10.1016/S0160-2896(89)80006-6

Lynn, R., & Irwing, P. (2004). Sex differences on the progressive matrices: A meta-analysis. *Intelligence*, *32*(5), 481-498. https://doi.org/10.1016/j.intell.2040.06.008

Lynn, R., & Irwing, P. (2008). Sex differences in mental arithmetic, digit span, and g defined as working memory capacity. *Intelligence*, *36*(3), 226-235. https://doi.org/10.1016/j.intell.2007.06.002

McDaniel, M. A. (2005). Big-brained people are smarter: A meta-analysis of the relationship between in vivo brain volume and intelligence. *Intelligence*, *33*(4), 337-346. https://doi.org/10.1016/j.intell.2004.11.005

Mukunda, K., & Hall, V. (1992). Does Performance on Memory for Order Correlate with Performance on Standardized Measures of Ability - a Metaanalysis. *Intelligence*, *16*(1), 81-97. https://doi.org/10.1016/0160-2896(92)90026-N

Murphy, N. A., & Hall, J. A. (2011). Intelligence and interpersonal sensitivity: A meta-analysis. *Intelligence*, *39*(1), 54-63. https://doi.org/10.1016/j.intell.2010.10.001

Paessler, K., Beinicke, A., & Hell, B. (2015). Interests and intelligence: A meta-analysis. *Intelligence*, *50*, 30-51. https://doi.org/10.1016/j.intell.2015.02.001

Pietschnig, J., & Gittler, G. (2017). Is ability-based emotional intelligence impervious to the Flynn effect? A cross-temporal meta-analysis (2001-2015). *Intelligence*, *61*, 37-45. https://doi.org/10.1016/j.intell.2016.12.006

Pietschnig, J., Voracek, M., & Formann, A. K. (2010). Mozart effect-Shmozart effect: A meta-analysis. *Intelligence*, *38*(3), 314-323. https://doi.org/10.1016/j.intell.2010.03.001

Roth, B., Becker, N., Romeyke, S., Schaefer, S., Domnick, F., & Spinath, F. M. (2015). Intelligence and school grades: A meta-analysis. *Intelligence*, *53*, 118-137. https://doi.org/10.1016/j.intell.2015.09.002

Sala, G., Burgoyne, A. P., Macnamara, B. N., Hambrick, D. Z., Campitelli, G., & Gobet, F. (2017). Checking the “Academic Selection” argument. Chess players outperform non-chess players in cognitive skills related to intelligence: A meta-analysis. *Intelligence*, *61*, 130-139. https://doi.org/10.1016/j.intell.2017.01.013

Shamosh, N. A., & Gray, J. R. (2008). Delay discounting and intelligence: A meta-analysis. *Intelligence*, *36*(4), 289-305. https://doi.org/10.1016/j.intell.2007.09.004

Stadler, M., Becker, N., Goedker, M., Leutner, D., & Greiff, S. (2015). Complex problem solving and intelligence: A meta-analysis. *Intelligence*, *53*, 92-101. https://doi.org/10.1016/j.intell.2015.09.005

Strenze, T. (2007). Intelligence and socioeconomic success: A meta-analytic review of longitudinal research. *Intelligence*, *35*(5), 401-426. https://doi.org/10.1016/j.intell.2006.09.004

Verive, J. M., & McDaniel, M. A. (1996). Short-term memory tests in personnel selection: Low adverse impact and high validity. *Intelligence*, *23*(1), 15-32. https://doi.org/10.1016/S0160-2896(96)80003-1

Webster, G. D., & Duffy, R. D. (2016). Losing faith in the intelligence-religiosity link: New evidence for a decline effect, spatial dependence, and mediation by education and life quality. *Intelligence*, *55*, 15-27. https://doi.org/10.1016/j.intell.2016.01.001

**Primary Studies**

Birenbaum, M., Kelly, A., & Levikeren, M. (1994). Stimulus Features and Sex-Differences in Mental Rotation Test-Performance. *Intelligence*, *19*(1), 51-64. https://doi.org/10.1016/0160-2896(94)90053-1

Colom, R., Juan-Espinosa, M., Abad, F., & Garcia, L. F. (2000). Negligible sex differences in general intelligence. *Intelligence*, *28*(1), 57-68. https://doi.org/10.1016/S0160-2896(99)00035-5

Delgado, A. R., & Prieto, G. (1997). Mental rotation as a mediator for sex-related differences in visualization. *Intelligence*, *24*(3), 405-416. https://doi.org/10.1016/S0160-2896(97)90057-X

Geiser, C., Lehmann, W., & Eid, M. (2008). A note on sex differences in mental rotation in different age groups. *Intelligence*, *36*(6), 556-563. https://doi.org/10.1016/j.intell.2007.12.003

Hausmann, M. (2014). Arts versus science - Academic background implicitly activates gender stereotypes on cognitive abilities with threat raising men’s (but lowering women’s) performance. *Intelligence*, *46*, 235-245. https://doi.org/10.1016/j.intell.2014.07.004

Hegarty, M., Montello, D. R., Richardson, A. E., Ishikawa, T., & Lovelace, K. (2006). Spatial abilities at different scales: Individual differences in aptitude-test performance and spatial-layout learning. *Intelligence*, *34*(2), 151-176. https://doi.org/10.1016/j.intell.2005.09.005

Jausovec, N., & Jausovec, K. (2008). Spatial rotation and recognizing emotions: Gender related differences in brain activity. *Intelligence*, *36*(5), 383-393. https://doi.org/10.1016/j.intell.2007.09.002

Ludeke, S. G., Rasmussen, S. H. R., & DeYoung, C. G. (2017). Verbal ability drives the link between intelligence and ideology in two American community samples. *Intelligence*, *61*, 1-6. https://doi.org/10.1016/j.intell.2016.10.006

Kaufman, S. B. (2007). Sex differences in mental rotation and spatial visualization ability: Can they be accounted for by differences in working memory capacity? *Intelligence*, *35*(3), 211-223. https://doi.org/10.1016/j.intell.2006.07.009

Keith, T. Z., Reynolds, M. R., Patel, P. G., & Ridley, K. P. (2008). Sex differences in latent cognitive abilities ages 6 to 59: Evidence from the Woodcock-Johnson III tests of cognitive abilities. *Intelligence*, *36*(6), 502-525. https://doi.org/10.1016/j.intell.2007.11.001

Lim, T. (1994). Gender-Related Differences in Intelligence - Application of Confirmatory Factor-Analysis. *Intelligence*, *19*(2), 179-192. https://doi.org/10.1016/0160-2896(94)90012-4

Lubinski, D., & Humphreys, L. (1990). A Broadly Based Analysis of Mathematical Giftedness. *Intelligence*, *14*(3), 327-355. https://doi.org/10.1016/0160-2896(90)90022-L

Majeres, R. L. (2007). Sex differences in phonological coding: Alphabet transformation speed. *Intelligence*, *35*(4), 335-346. https://doi.org/10.1016/j.intell.2006.08.005

Moe, A. (2016). Teaching motivation and strategies to improve mental rotation abilities. *Intelligence*, *59*, 16-23. https://doi.org/10.1016/j.intell.2016.10.004

Reilly, D., Neumann, D. L., & Andrews, G. (2016). Sex and sex-role differences in specific cognitive abilities. *Intelligence*, *54*, 147-158. https://doi.org/10.1016/j.intell.2015.12.004

Rojahn, J., & Naglieri, J. A. (2006). Developmental gender differences on the Naglieri Nonverbal Ability Test in a nationally normed sample of 5-17 year olds. *Intelligence*, *34*(3), 253-260. https://doi.org/10.1016/j.intell.2005.09.004

Sommer, M., & Arendasy, M. E. (2014). Comparing different explanations of the effect of test anxiety on respondents’ test scores. *Intelligence*, *42*, 115-127. https://doi.org/10.1016/j.intell.2013.11.003

Sommer, M., & Arendasy, M. E. (2015). Further evidence for the deficit account of the test anxiety-test performance relationship from a high-stakes admission testing setting. *Intelligence*, *53*, 72-80. https://doi.org/10.1016/j.intell.2015.08.007

te Nijenhuis, J., Resing, W., Tolboom, E., & Bleichrodt, N. (2004). Short-term memory as an additional predictor of school achievement for immigrant children? *Intelligence*, *32*(2), 203-213. https://doi.org/10.1016/j.intell.2003.07.001

Toivainen, T., Papageorgiou, K. A., Tosto, M. G., & Kovas, Y. (2017). Sex differences in non-verbal and verbal abilities in childhood and adolescence. *Intelligence*, *64*, 81-88. https://doi.org/10.1016/j.intell.2017.07.007

Turkheimer, E., Farace, E., Yeo, R., & Bigler, E. (1993). Quantitative-Analysis of Gender Differences in the Effects of Lateralized Lesions on Verbal and Performance IQ. *Intelligence*, *17*(4), 461-474. https://doi.org/10.1016/0160-2896(93)90013-U

Vederhus, L., & Krekling, S. (1996). Sex differences in visual spatial ability in 9-year-old children. *Intelligence*, *23*(1), 33-43. https://doi.org/10.1016/S0160-2896(96)80004-3

Yeo, R. A., Ryman, S. G., Pommy, J., Thoma, R. J., & Jung, R. E. (2016). General cognitive ability and fluctuating asymmetry of brain surface area. *Intelligence*, *56*, 93-98. https://doi.org/10.1016/j.intell.2016.03.002

**Conceptual Paper**

Santarnecchi, E., Emmendorfer, A., Tadayon, S., Rossi, S., Rossi, A., & Pascual-Leone, A. (2017). Network connectivity correlates of variability in fluid intelligence performance. *Intelligence*, *65*, 35-47. https://doi.org/10.1016/j.intell.2017.10.002

**Archival Studies**

Deary, I. J., Irwing, P., Der, G., & Bates, T. C. (2007). Brother-sister differences in the g factor in intelligence: Analysis of full, opposite-sex siblings from the NLSY 1979. *Intelligence*, *35*(5), 451-456. https://doi.org/10.1016/j.intell.2006.09.003

Stoet, G., & Geary, D. C. (2015). Sex differences in academic achievement are not related to political, economic, or social equality. *Intelligence*, *48*, 137-151. https://doi.org/10.1016/j.intell.2014.11.006

**Commentary**

Dodonova, Y. A., & Dodonov, Y. S. (2013). Is there any evidence of historical slowing of reaction time? No, unless we compare apples and oranges. *Intelligence*, *41*(5), 674-687. https://doi.org/10.1016/j.intell.2013.09.001

**No Traditional Meta-Analytical Estimate**

Armstrong, E. L., Woodley, M. A., & Lynn, R. (2014). Cognitive abilities amongst the Sami population. *Intelligence*, *46*, 35-39. https://doi.org/10.1016/j.intell.2014.03.009

Basten, U., Hilger, K., & Fiebach, C. J. (2015). Where smart brains are different: A quantitative meta-analysis of functional and structural brain imaging studies on intelligence. *Intelligence*, *51*, 10-27. https://doi.org/10.1016/j.intell.2015.04.009

Blum, D., & Holling, H. (2017). Spearman’s law of diminishing returns. A meta-analysis. *Intelligence*, *65*, 60-66. https://doi.org/10.1016/j.intell.2017.07.004

Fernandes, H. B. F., Woodley, M. A., & te Nijenhuis, J. (2014). Differences in cognitive abilities among primates are concentrated on G: Phenotypic and phylogenetic comparisons with two meta-analytical databases. *Intelligence*, *46*, 311-322. https://doi.org/10.1016/j.intell.2014.07.007

Flynn, J. R., te Nijenhuis, J., & Metzen, D. (2014). The g beyond Spearman’s g: Flynn’s paradoxes resolved using four exploratory meta-analyses. *Intelligence*, *44*, 1-10. https://doi.org/10.1016/j.intell.2014.01.009

Kura, K., Armstrong, E. L., & Templer, D. I. (2014). Cognitive function among the Ainu people. *Intelligence*, *44*, 149-154. https://doi.org/10.1016/j.intell.2014.04.001

Lee, J. J. (2007). A g beyond Homo sapiens? Some hints and suggestions. *Intelligence*, *35*(3), 253-265. https://doi.org/10.1016/j.intell.2006.08.003

Pietschnig, J., & Gittler, G. (2015). A reversal of the Flynn effect for spatial perception in German-speaking countries: Evidence from a cross-temporal IRT-based meta-analysis (1977-2014). *Intelligence*, *53*, 145-153. https://doi.org/10.1016/j.intell.2015.10.004

Protzko, J. (2015). The environment in raising early intelligence: A meta-analysis of the fadeout effect. *Intelligence*, *53*, 202-210. https://doi.org/10.1016/j.intell.2015.10.006

Santarnecchi, E., Emmendorfer, A., & Pascual-Leone, A. (2017). Dissecting the parieto-frontal correlates of fluid intelligence: A comprehensive ALE meta-analysis study. *Intelligence*, *63*, 9-28. https://doi.org/10.1016/j.intell.2017.04.008

Scharfen, J., Peters, J. M., & Holling, H. (2018). Retest effects in cognitive ability tests: A meta-analysis. *Intelligence*, *67*, 44-66. https://doi.org/10.1016/j.intell.2018.01.003

Sprugnoli, G., Rossi, S., Emmendorfer, A., Rossi, A., Liew, S.-L., Tatti, E., … Santarnecchi, E. (2017). Neural correlates of Eureka moment. *Intelligence*, *62*, 99-118. https://doi.org/10.1016/j.intell.2017.03.004

te Nijenhuis, J., & van der Flier, H. (2013). Is the Flynn effect on g?: A meta-analysis. *Intelligence*, *41*(6), 802-807. https://doi.org/10.1016/j.intell.2013.03.001

te Nijenhuis, J., David, H., Metzen, D., & Armstrong, E. L. (2014). Spearman’s hypothesis tested on European Jews vs non-Jewish Whites and vs Oriental Jews: Two meta-analyses. *Intelligence*, *44*, 15-18. https://doi.org/10.1016/j.intell.2014.02.002

te Nijenhuis, J., Jongeneel-Grimen, B., & Kirkegaard, E. O. W. (2014). Are Headstart gains on the g factor? A meta-analysis. *Intelligence*, *46*, 209-215. https://doi.org/10.1016/j.intell.2014.07.001

te Nijenhuis, J., Kura, K., & Hur, Y.-M. (2014). The correlation between g loadings and heritability in Japan: A meta-analysis. *Intelligence*, *46*, 275-282. https://doi.org/10.1016/j.intell.2014.07.008

te Nijenhuis, J., van den Hoek, M., & Armstrong, E. L. (2015). Spearman’s hypothesis and Amerindians: A meta-analysis. *Intelligence*, *50*, 87-92. https://doi.org/10.1016/j.intell.2015.02.006

te Nijenhuis, J., van Vianen, A. E. M., & van der Flier, H. (2007). Score gains on g-loaded tests: No g. *Intelligence*, *35*(3), 283-300. https://doi.org/10.1016/j.intell.2006.07.006

te Nijenhuis, J., Willigers, D., Dragt, J., & van der Flier, H. (2016). The effects of language bias and cultural bias estimated using the method of correlated vectors on a large database of IQ comparisons between native Dutch and ethnic minority immigrants from non-Western countries. *Intelligence*, *54*, 117-135. https://doi.org/10.1016/j.intell.2015.12.003

Woodley, M. A., te Nijenhuis, J., & Murphy, R. (2013). Were the Victorians cleverer than us? The decline in general intelligence estimated from a meta-analysis of the slowing of simple reaction time. *Intelligence*, *41*(6), 843-850. <https://doi.org/10.1016/j.intell.2013.04.006>

Woodley, M. A., te Nijenhuis, J., & Murphy, R. (2014). Is there a dysgenic secular trend towards slowing simple reaction time? Responding to a quartet of critical commentaries. *Intelligence*, *46*, 131-147. https://doi.org/10.1016/j.intell.2014.05.012

Woodley, M. A., te Nijenhuis, J., Must, O., & Must, A. (2014). Controlling for increased guessing enhances the independence of the Flynn effect from g: The return of the Brand effect. *Intelligence*, *43*, 27-34. https://doi.org/10.1016/j.intell.2013.12.004

Wongupparaj, P., Kumari, V., & Morris, R. G. (2015). A Cross-Temporal Meta-Analysis of Raven’s Progressive Matrices: Age groups and developing versus developed countries. *Intelligence*, *49*, 1-9. https://doi.org/10.1016/j.intell.2014.11.008

Wongupparaj, P., Wongupparaj, R., Kumari, V., & Morris, R. G. (2017). The Flynn effect for verbal and visuospatial short-term and working memory: A cross-temporal meta-analysis. *Intelligence*, *64*, 71-80. https://doi.org/10.1016/j.intell.2017.07.006

**Initial Study not Identifiable / Retrievable**

Burgoyne, A. P., Sala, G., Gobet, F., Macnamara, B. N., Campitelli, G., & Hambrick, D. Z. (2016). The relationship between cognitive ability and chess skill: A comprehensive meta-analysis. *Intelligence*, *59*, 72-83. https://doi.org/10.1016/j.intell.2016.08.002

Grove, B. J., Lim, S. J., Gale, C. R., & Shenkin, S. D. (2017). Birth weight and cognitive ability in adulthood: A systematic review and meta-analysis. *Intelligence*, *61*, 146-158. https://doi.org/10.1016/j.intell2017.02.001
