# Supplementary material for: Effect Declines Are Systematic, Strong, and Ubiquitous: A Meta-Meta-Analysis of the Decline Effect in Intelligence Research
Source: Front Psychol. 2019 Dec 19;10:2874. doi: 10.3389/fpsyg.2019.02874 (PMC6930891; doi:10.3389/fpsyg.2019.02874)
Supplement: Supplementary file 4 [file Data_Sheet_4.docx]

library(foreign)

library(metafor)

library(pwr)

library(car)

library(psych)

#############################

## open with S3_data.csv with SPSS and save as .sav file ##

## data1 = S3_data.sav ##

## define as data.frame ##

## do not use value labels ##

#############################

## data1<-read.spss

rdiff<-fisherz2r(data1$abs_crude_difference)

rslope<-fisherz2r(data1$abs_slope_z_self)

data2<-cbind(data1,rdiff,rslope)

##crude differences overall

##RE

rma(ri=data2$rdiff,ni=data2$k-3,measure="ZCOR",control=list(stepadj=0.5))

sum(data2$n)

##FE model

rma(ri=data2$rdiff,ni=data2$k-3,measure="ZCOR",method="FE")

##crude differences decline

decline<-subset(data2,data2$direction_incr_decl=="decline")

##RE

sum(decline$n)

rma(ri=decline$rdiff,ni=decline$k-3,measure="ZCOR",control=list(stepadj=0.5))

##FE model

rma(ri=decline$rdiff,ni=decline$k-3,measure="ZCOR",method="FE")

##crude differences increase

increase<-subset(data2,data2$direction_incr_decl=="increase")

##RE

sum(increase$n)

rma(ri=increase$rdiff,ni=increase$k-3,measure="ZCOR",control=list(stepadj=0.5))

##FE model

rma(ri=increase$rdiff,ni=increase$k-3,measure="ZCOR",method="FE")

##crude differences proteus

proteus<-subset(data2,data2$direction_incr_decl=="proteus")

##RE

sum(proteus$n)

rma(ri=proteus$rdiff,ni=proteus$k-3,measure="ZCOR",control=list(stepadj=0.5))

##FE model

rma(ri=proteus$rdiff,ni=proteus$k-3,measure="ZCOR",method="FE")

#####

## Meta-regressions OVERALL

##mixed effects initial n

regr1<-rma(ri=data2$rdiff,ni=data2$k,mods=~data2$n_initial_study,measure="ZCOR",control=list(stepadj=0.5))

regr1

sum(data2$n[which(data2$n_initial_study>1)])

##FE-regression

lm1<-lm(data2$rdiff~data2$n_initial_study,weights=data2$k-3)

summary(lm1)

etasq(lm1)

##unweighted

lm1.1<-lm(data2$rdiff~data2$n_initial_study)

summary(lm1.1)

etasq(lm1.1)

##mixed effects summary effect

regr2<-rma(ri=data2$rdiff,ni=data2$k,mods=~abs(data2$MA_ES_z),measure="ZCOR",control=list(stepadj=0.5))

regr2

sum(data2$n[which(data2$MA_ES_z>-2)])

##FE-regression

lm2<-lm(data2$rdiff~abs(data2$MA_ES_z),weights=data2$k-3)

summary(lm2)

etasq(lm2)

##unweighted

lm1.2<-lm(data2$rdiff~abs(data2$MA_ES_z))

summary(lm1.2)

etasq(lm1.2)

##mixed effects initial effect

regr3<-rma(ri=data2$rdiff,ni=data2$k,mods=~abs(data2$initial_ES_z),measure="ZCOR",control=list(stepadj=0.5))

regr3

sum(data2$n[which(data2$initial_ES_z>-2)])

##FE-regression

lm3<-lm(data2$rdiff~abs(data2$initial_ES_z),weights=data2$k-3)

summary(lm3)

etasq(lm3)

##unweighted

lm1.3<-lm(data2$rdiff~abs(data2$initial_ES_z))

summary(lm1.3)

etasq(lm1.3)

##mixed effects IF

regr4<-rma(ri=data2$rdiff,ni=data2$k,mods=~data2$IF_initial_aktuell,measure="ZCOR",control=list(stepadj=0.5))

regr4

sum(data2$n[which(data2$IF_initial_aktuell>-2)])

##FE-regression

lm4<-lm(data2$rdiff~data2$IF_initial_aktuell,weights=data2$k-3)

summary(lm4)

etasq(lm4)

##unweighted

lm1.4<-lm(data2$rdiff~data2$IF_initial_aktuell)

summary(lm1.4)

etasq(lm1.4)

##mixed effects citation numbers (annual)

regr6<-rma(ri=data2$rdiff,ni=data2$k,mods=~data2$winsor_annual_citations,measure="ZCOR",control=list(stepadj=0.5))

regr6

sum(data2$n[which(data2$winsor_annual_citations>-2)])

##FE-regression

lm6<-lm(data2$rdiff~data2$winsor_annual_citations,weights=data2$k-3)

summary(lm6)

etasq(lm6)

##unweighted

lm1.6<-lm(data2$rdiff~data2$winsor_annual_citations)

summary(lm1.6)

etasq(lm1.6)

## Meta-regressions DECLINE

##mixed effects initial n

regr1<-rma(ri=decline$rdiff,ni=decline$k,mods=~decline$n_initial_study,measure="ZCOR",control=list(stepadj=0.5))

regr1

sum(data2$n[which(decline$n_initial_study>1)])

##FE-regression

lm1<-lm(decline$rdiff~decline$n_initial_study,weights=decline$k-3)

summary(lm1)

etasq(lm1)

##unweighted

lm1.1<-lm(decline$rdiff~decline$n_initial_study)

summary(lm1.1)

etasq(lm1.1)

##mixed effects summary effect

regr2<-rma(ri=decline$rdiff,ni=decline$k,mods=~abs(decline$MA_ES_z),measure="ZCOR",control=list(stepadj=0.5))

regr2

sum(data2$n[which(decline$MA_ES_z>-10)])

##FE-regression

lm2<-lm(decline$rdiff~abs(decline$MA_ES_z),weights=decline$k-3)

summary(lm2)

etasq(lm2)

##unweighted

lm1.2<-lm(decline$rdiff~abs(decline$MA_ES_z))

summary(lm1.2)

etasq(lm1.2)

##mixed effects initial effect

regr3<-rma(ri=decline$rdiff,ni=decline$k,mods=~abs(decline$initial_ES_z),measure="ZCOR",control=list(stepadj=0.5))

regr3

sum(data2$n[which(decline$initial_ES_z>-10)])

##FE-regression

lm3<-lm(decline$rdiff~abs(decline$initial_ES_z),weights=decline$k-3)

summary(lm3)

etasq(lm3)

##unweighted

lm1.3<-lm(decline$rdiff~abs(decline$initial_ES_z))

summary(lm1.3)

etasq(lm1.3)

##mixed effects IF

regr4<-rma(ri=decline$rdiff,ni=decline$k,mods=~decline$IF_initial_aktuell,measure="ZCOR",control=list(stepadj=0.5))

regr4

sum(decline$n[which(decline$IF_initial_aktuell>-1)])

##FE-regression

lm4<-lm(decline$rdiff~decline$IF_initial_aktuell,weights=decline$k-3)

summary(lm4)

etasq(lm4)

##unweighted

lm1.4<-lm(decline$rdiff~decline$IF_initial_aktuell)

summary(lm1.4)

etasq(lm1.4)

##mixed effects citation numbers (annual)

regr6<-rma(ri=decline$rdiff,ni=decline$k,mods=~decline$winsor_annual_citations,measure="ZCOR",control=list(stepadj=0.5))

regr6

sum(decline$n[which(decline$winsor_annual_citations>-1)])

##FE-regression

lm6<-lm(decline$rdiff~decline$winsor_annual_citations,weights=decline$k-3)

summary(lm6)

etasq(lm6)

##unweighted

lm1.6<-lm(decline$rdiff~decline$winsor_annual_citations)

summary(lm1.6)

etasq(lm1.6)

##coefficients overall

##RE

##rma(ri=data2$rdiff,ni=data2$k,measure="ZCOR",control=list(stepadj=0.5))

sum(data2$n[which(data2$rslope>-10)])

rma(ri=data2$rslope,ni=data2$k-3,measure="ZCOR")

##FE model

##rma(ri=data2$rdiff,ni=data2$k,measure="ZCOR",method="FE")

rma(ri=data2$rslope,ni=data2$k-3,measure="ZCOR",method="FE")

##coefficients decline

sum(decline$n[which(decline$rslope>-1)])

decline<-subset(data2,data2$direction_incr_decl=="decline")

##RE

rma(ri=decline$rslope,ni=decline$k-3,measure="ZCOR")

##FE model

rma(ri=decline$rslope,ni=decline$k-3,measure="ZCOR",method="FE")

##coefficients increase

increase<-subset(data2,data2$direction_incr_decl=="increase")

sum(increase$n[which(increase$rslope>-1)])

##RE

rma(ri=increase$rslope,ni=increase$k-3,measure="ZCOR")

##FE model

rma(ri=increase$rslope,ni=increase$k-3,measure="ZCOR",method="FE")

##coefficients proteus

proteus<-subset(data2,data2$direction_incr_decl=="proteus")

sum(proteus$n[which(proteus$rslope>-1)])

##RE

rma(ri=proteus$rslope,ni=proteus$k-3,measure="ZCOR")

##FE model

rma(ri=proteus$rslope,ni=proteus$k-3,measure="ZCOR",method="FE")

#####

## Meta-regressions OVERALL - coefficient

##mixed effects initial n

regr1<-rma(ri=data2$rslope,ni=data2$k,mods=~data2$n_initial_study,measure="ZCOR",control=list(stepadj=0.5))

regr1

sum(data2$n[which(data2$n_initial_study>1 & data2$rslope>-10)])

##FE-regression

lm1<-lm(data2$rslope~data2$n_initial_study,weights=data2$k-3)

summary(lm1)

etasq(lm1)

##unweighted

lm1.1<-lm(data2$rslope~data2$n_initial_study)

summary(lm1.1)

etasq(lm1.1)

##mixed effects summary effect

regr2<-rma(ri=data2$rslope,ni=data2$k,mods=~abs(data2$MA_ES_z),measure="ZCOR",control=list(stepadj=0.5))

regr2

sum(data2$n[which(data2$MA_ES_z>-5 & data2$rslope>-10)])

##FE-regression

lm2<-lm(data2$rslope~abs(data2$MA_ES_z),weights=data2$k-3)

summary(lm2)

etasq(lm2)

##unweighted

lm1.2<-lm(data2$rslope~abs(data2$MA_ES_z))

summary(lm1.2)

etasq(lm1.2)

##mixed effects initial effect

regr3<-rma(ri=data2$rslope,ni=data2$k,mods=~abs(data2$initial_ES_z),measure="ZCOR",control=list(stepadj=0.5))

regr3

sum(data2$n[which(data2$initial_ES_z>-5 & data2$rslope>-10)])

##FE-regression

lm3<-lm(data2$rslope~abs(data2$initial_ES_z),weights=data2$k-3)

summary(lm3)

etasq(lm3)

##unweighted

lm1.3<-lm(data2$rslope~abs(data2$initial_ES_z))

summary(lm1.3)

etasq(lm1.3)

##mixed effects IF

regr4<-rma(ri=data2$rslope,ni=data2$k,mods=~data2$IF_initial_aktuell,measure="ZCOR",control=list(stepadj=0.5))

regr4

sum(data2$n[which(data2$IF_initial_aktuell>-5 & data2$rslope>-10)])

##FE-regression

lm4<-lm(data2$rslope~data2$IF_initial_aktuell,weights=data2$k-3)

summary(lm4)

etasq(lm4)

##unweighted

lm1.4<-lm(data2$rslope~data2$IF_initial_aktuell)

summary(lm1.4)

etasq(lm1.4)

##mixed effects citation numbers (annual)

regr6<-rma(ri=data2$rslope,ni=data2$k,mods=~data2$winsor_annual_citations,measure="ZCOR",control=list(stepadj=0.5))

regr6

sum(data2$n[which(data2$winsor_annual_citations>-5 & data2$rslope>-10)])

##FE-regression

lm6<-lm(data2$rslope~data2$winsor_annual_citations,weights=data2$k-3)

summary(lm6)

etasq(lm6)

##unweighted

lm1.6<-lm(data2$rslope~data2$winsor_annual_citations)

summary(lm1.6)

etasq(lm1.6)

## Meta-regressions DECLINE - coefficient

##mixed effects initial n

regr1<-rma(ri=decline$rslope,ni=decline$k,mods=~decline$n_initial_study,measure="ZCOR",control=list(stepadj=0.5))

regr1

sum(decline$n[which(decline$n_initial_study>1 & decline$rslope>-10)])

##FE-regression

lm1<-lm(decline$rslope~decline$n_initial_study,weights=decline$k-3)

summary(lm1)

etasq(lm1)

##unweighted

lm1.1<-lm(decline$rslope~decline$n_initial_study)

summary(lm1.1)

etasq(lm1.1)

##mixed effects summary effect

regr2<-rma(ri=decline$rslope,ni=decline$k,mods=~abs(decline$MA_ES_z),measure="ZCOR",control=list(stepadj=0.5))

regr2

sum(decline$n[which(decline$MA_ES_z>-10 & decline$rslope>-10)])

##FE-regression

lm2<-lm(decline$rslope~abs(decline$MA_ES_z),weights=decline$k-3)

summary(lm2)

etasq(lm2)

##unweighted

lm1.2<-lm(decline$rslope~abs(decline$MA_ES_z))

summary(lm1.2)

etasq(lm1.2)

##mixed effects initial effect

regr3<-rma(ri=decline$rslope,ni=decline$k,mods=~abs(decline$initial_ES_z),measure="ZCOR",control=list(stepadj=0.5))

regr3

sum(decline$n[which(decline$initial_ES_z>-10 & decline$rslope>-10)])

##FE-regression

lm3<-lm(decline$rslope~abs(decline$initial_ES_z),weights=decline$k-3)

summary(lm3)

etasq(lm3)

##unweighted

lm1.3<-lm(decline$rslope~abs(decline$initial_ES_z))

summary(lm1.3)

etasq(lm1.3)

##mixed effects IF

regr4<-rma(ri=decline$rslope,ni=decline$k,mods=~decline$IF_initial_aktuell,measure="ZCOR",control=list(stepadj=0.5))

regr4

sum(decline$n[which(decline$IF_initial_aktuell>-10 & decline$rslope>-10)])

##FE-regression

lm4<-lm(decline$rslope~decline$IF_initial_aktuell,weights=decline$k-3)

summary(lm4)

etasq(lm4)

##unweighted

lm1.4<-lm(decline$rslope~decline$IF_initial_aktuell)

summary(lm1.4)

etasq(lm1.4)

##mixed effects citation numbers (annual)

regr6<-rma(ri=decline$rslope,ni=decline$k,mods=~decline$winsor_annual_citations,measure="ZCOR",control=list(stepadj=0.5))

regr6

sum(decline$n[which(decline$winsor_annual_citations>-10 & decline$rslope>-10)])

##FE-regression

lm6<-lm(decline$rslope~decline$winsor_annual_citations,weights=decline$k-3)

summary(lm6)

etasq(lm6)

##unweighted

lm1.6<-lm(decline$rslope~decline$winsor_annual_citations)

summary(lm1.6)

etasq(lm1.6)

###############

## I-Squared ##

###############

##overall

mean(data2$I_squared_self,na.rm=T)

##decline

mean(decline$I_squared_self,na.rm=T)

###################

## influence of initial study power

##overall

##mixed effects power

regr.pow<-rma(ri=data2$rdiff,ni=data2$k,mods=~data2$init_pow,measure="ZCOR",control=list(stepadj=0.5))

regr.pow

sum(data2$n[which(data2$init_pow>.0001)])

##FE-regression

lm1<-lm(data2$rdiff~data2$init_pow,weights=data2$k-3)

summary(lm1)

etasq(lm1)

##unweighted

lm1.1<-lm(data2$rdiff~data2$init_pow)

summary(lm1.1)

etasq(lm1.1)

## decline

##mixed effects power

regr.pow<-rma(ri=decline$rdiff,ni=decline$k,mods=~decline$init_pow,measure="ZCOR",control=list(stepadj=0.5))

regr.pow

sum(data2$n[which(decline$init_pow>0.0001)])

##FE-regression

lm1<-lm(decline$rdiff~decline$init_pow,weights=decline$k-3)

summary(lm1)

etasq(lm1)

##unweighted

lm1.1<-lm(decline$rdiff~decline$init_pow)

summary(lm1.1)

etasq(lm1.1)

###################

## influence of initial publication year

##Crude

##overall

regr.year<-rma(ri=data2$rdiff,ni=data2$k,mods=~data2$firstyear,measure="ZCOR",control=list(stepadj=0.5))

regr.year

sum(data2$n[which(data2$firstyear>1)])

##FE-regression

lm1<-lm(data2$rdiff~data2$firstyear,weights=data2$k-3)

summary(lm1)

etasq(lm1)

##unweighted

lm1.1<-lm(data2$rdiff~data2$firstyear)

summary(lm1.1)

etasq(lm1.1)

## decline

##mixed effects publication year

regr.year<-rma(ri=decline$rdiff,ni=decline$k,mods=~decline$firstyear,measure="ZCOR",control=list(stepadj=0.5))

regr.year

sum(data2$n[which(decline$firstyear>1)])

##FE-regression

lm1<-lm(decline$rdiff~decline$firstyear,weights=decline$k-3)

summary(lm1)

etasq(lm1)

##unweighted

lm1.1<-lm(decline$rdiff~decline$firstyear)

summary(lm1.1)

etasq(lm1.1)

##Slope

## overall

##mixed effects publication year

regr1<-rma(ri=data2$rslope,ni=data2$k,mods=~data2$firstyear,measure="ZCOR",control=list(stepadj=0.5))

regr1

sum(data2$n[which(data2$firstyear>1 & data2$rslope>-10)])

##FE-regression

lm1<-lm(data2$rslope~data2$firstyear,weights=data2$k-3)

summary(lm1)

etasq(lm1)

##unweighted

lm1.1<-lm(data2$rslope~data2$firstyear)

summary(lm1.1)

etasq(lm1.1)

##decline

##mixed effects publication year

regr1<-rma(ri=decline$rslope,ni=decline$k,mods=~decline$firstyear,measure="ZCOR",control=list(stepadj=0.5))

regr1

sum(decline$n[which(decline$firstyear>1 & decline$rslope>-10)])

##FE-regression

lm1<-lm(decline$rslope~decline$firstyear,weights=decline$k-3)

summary(lm1)

etasq(lm1)

##unweighted

lm1.1<-lm(decline$rslope~decline$firstyear)

summary(lm1.1)

etasq(lm1.1)

########################

## Regressions with winsorized annual citation year

##crude

##mixed effects citation numbers (annual)

regr6<-rma(ri=data2$rdiff,ni=data2$k,mods=~data2$winsor_annual_citations,measure="ZCOR",control=list(stepadj=0.5))

regr6

sum(data2$n[which(data2$winsor_annual_citations>-2)])

##FE-regression

lm6<-lm(data2$rdiff~data2$winsor_annual_citations,weights=data2$k-3)

summary(lm6)

etasq(lm6)

##unweighted

lm1.6<-lm(data2$rdiff~data2$winsor_annual_citations)

summary(lm1.6)

etasq(lm1.6)

##mixed effects citation numbers (annual)

regr6<-rma(ri=decline$rdiff,ni=decline$k,mods=~decline$winsor_annual_citations,measure="ZCOR",control=list(stepadj=0.5))

regr6

sum(decline$n[which(decline$winsor_annual_citations>-1)])

##FE-regression

lm6<-lm(decline$rdiff~decline$winsor_annual_citations,weights=decline$k-3)

summary(lm6)

etasq(lm6)

##unweighted

lm1.6<-lm(decline$rdiff~decline$winsor_annual_citations)

summary(lm1.6)

etasq(lm1.6)

##regression

##mixed effects citation numbers (annual)

regr6<-rma(ri=data2$rslope,ni=data2$k,mods=~data2$winsor_annual_citations,measure="ZCOR",control=list(stepadj=0.5))

regr6

sum(data2$n[which(data2$winsor_annual_citations>-5 & data2$rslope>-10)])

##FE-regression

lm6<-lm(data2$rslope~data2$winsor_annual_citations,weights=data2$k-3)

summary(lm6)

etasq(lm6)

##unweighted

lm1.6<-lm(data2$rslope~data2$winsor_annual_citations)

summary(lm1.6)

etasq(lm1.6)

##mixed effects citation numbers (annual)

regr6<-rma(ri=decline$rslope,ni=decline$k,mods=~decline$winsor_annual_citations,measure="ZCOR",control=list(stepadj=0.5))

regr6

sum(decline$n[which(decline$winsor_annual_citations>-10 & decline$rslope>-10)])

##FE-regression

lm6<-lm(decline$rslope~decline$winsor_annual_citations,weights=decline$k-3)

summary(lm6)

etasq(lm6)

##unweighted

lm1.6<-lm(decline$rslope~decline$winsor_annual_citations)

summary(lm1.6)

etasq(lm1.6)
