# Supplementary figures and images for: Effect Declines Are Systematic, Strong, and Ubiquitous: A Meta-Meta-Analysis of the Decline Effect in Intelligence Research
Source: Front Psychol. 2019 Dec 19;10:2874. doi: 10.3389/fpsyg.2019.02874 (PMC6930891; doi:10.3389/fpsyg.2019.02874)

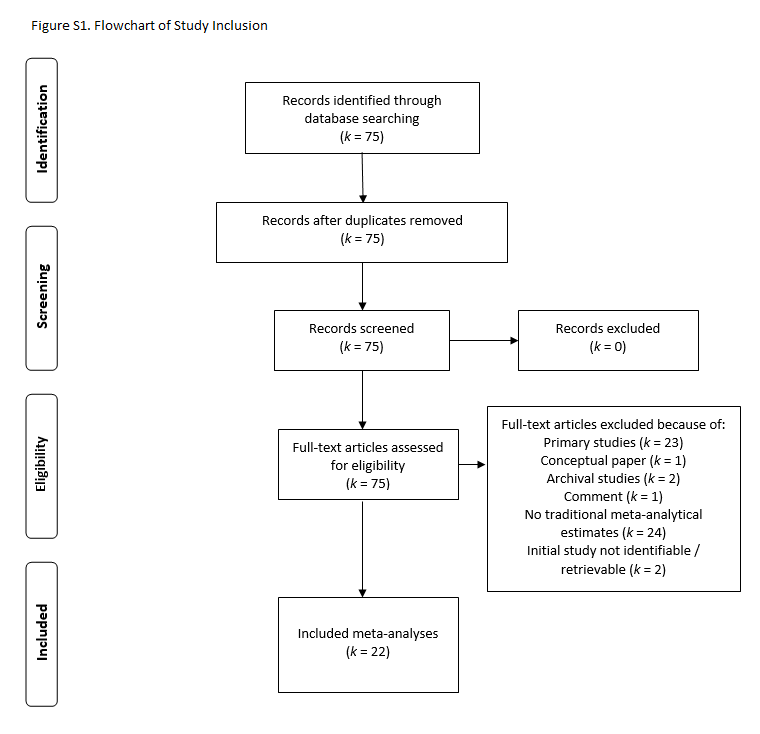

Supplement: Supplementary file 5 [file Image_1.TIF]
